# The Microbiota–Gut–Brain Axis and Neurological Disorders: A Comprehensive Review

**DOI:** 10.3390/life14101234

**Published:** 2024-09-26

**Authors:** Mohammed M. Nakhal, Lidya K. Yassin, Rana Alyaqoubi, Sara Saeed, Alreem Alderei, Alya Alhammadi, Mirah Alshehhi, Afra Almehairbi, Shaikha Al Houqani, Shamsa BaniYas, Haia Qanadilo, Bassam R. Ali, Safa Shehab, Yauhen Statsenko, Sarah Meribout, Bassem Sadek, Amal Akour, Mohammad I. K. Hamad

**Affiliations:** 1 Department of Anatomy, College of Medicine and Health Sciences, United Arab Emirates University, Al Ain P.O. Box 15551, United Arab Emirates202213584@uaeu.ac.ae (S.B.); s.shehab@uaeu.ac.ae (S.S.); 2 Department of Genetics and Genomics, College of Medicine and Health Sciences, United Arab Emirates University, Al Ain P.O. Box 15551, United Arab Emirates; bassam.ali@uaeu.ac.ae; 3 Department of Radiology, College of Medicine and Health Sciences, United Arab Emirates University, Al Ain P.O. Box 15551, United Arab Emirates; e.a.statsenko@uaeu.ac.ae; 4 Neuroscience Platform, ASPIRE Precision Medicine Institute in Abu Dhabi, United Arab Emirates University, Al Ain P.O. Box 15551, United Arab Emirates; 5 Internal Medicine Department, Maimonides Medical Center, New York, NY 11219, USA; smeribout18@gmail.com; 6 Department of Pharmacology & Therapeutics, College of Medicine and Health Sciences, United Arab Emirates University, Al Ain P.O. Bo Box 15551, United Arab Emirates; bassem.sadek@uaeu.ac.ae (B.S.); aakour@uaeu.ac.ae (A.A.); 7 Zayed Center for Health Sciences, United Arab Emirates University, Al Ain P.O. Box 1551, United Arab Emirates; 8 Department of Biopharmaceutics and Clinical Pharmacy, School of Pharmacy, The University of Jordan, Amman 11942, Jordan

**Keywords:** microbiota, microbiota–gut–brain axis, psychotropic agents, neurological disorders and neurodegenerative diseases

## Abstract

Microbes have inhabited the earth for hundreds of millions of years longer than humans. The microbiota–gut–brain axis (MGBA) represents a bidirectional communication pathway. These communications occur between the central nervous system (CNS), the enteric nervous system (ENS), and the emotional and cognitive centres of the brain. The field of research on the gut–brain axis has grown significantly during the past two decades. Signalling occurs between the gut microbiota and the brain through the neural, endocrine, immune, and humoral pathways. A substantial body of evidence indicates that the MGBA plays a pivotal role in various neurological diseases. These include Alzheimer’s disease (AD), autism spectrum disorder (ASD), Rett syndrome, attention deficit hyperactivity disorder (ADHD), non-Alzheimer’s neurodegeneration and dementias, fronto-temporal lobe dementia (FTLD), Wilson–Konovalov disease (WD), multisystem atrophy (MSA), Huntington’s chorea (HC), Parkinson’s disease (PD), multiple sclerosis (MS), amyotrophic lateral sclerosis (ALS), temporal lobe epilepsy (TLE), depression, and schizophrenia (SCZ). Furthermore, the bidirectional correlation between therapeutics and the gut–brain axis will be discussed. Conversely, the mood of delivery, exercise, psychotropic agents, stress, and neurologic drugs can influence the MGBA. By understanding the MGBA, it may be possible to facilitate research into microbial-based interventions and therapeutic strategies for neurological diseases.

## 1. Introduction

The gut microbiota, a vast collection of microorganisms residing in the gastrointestinal (GI) tract, has the ability to modulate brain function. Unlike the brain, the gut microbiota is highly accessible to direct interventions, such as prebiotics, probiotics, and antibiotics, and can be influenced by lifestyle. The concept of the MGBA emerged from extensive research demonstrating a clear connection between the gut and the brain; for review see [1,2,3]. A considerable body of research has demonstrated that the microbiota may play a role in brain morphology and function. For instance, germ-free (GF) animals have exhibited brain abnormalities in the absence of microbiota [4,5,6,7,8]. Furthermore, alterations in behaviour have been observed in animals administered specific strains of bacteria [9,10,11,12]. Furthermore, exposure to a single microbial strain has been demonstrated to protect against certain stress-induced behaviours and systemic immune alterations. This work supports the hypothesis that microbe-based interventions may be beneficial for the treatment of stress-related disorders [13]. These findings have also been observed in animal studies, where low-level infections have been shown to alter behaviour even in the absence of an overt immunologic response [14]. Furthermore, preclinical studies have demonstrated that the administration of antibiotics in early life, through the temporary disruption of the gut microbiota, results in specific and long-lasting changes in visceral sensitivity in rats [15].

The gut microbiota can communicate with the brain in various ways, including neuronal pathways and small molecule messaging systems. However, further research is necessary to fully comprehend the impact of bacteria in the GI tract on the brain and behaviour. Signals generated in the gut can be transmitted to the brain through various pathways [16,17]. The primary mode of immune communication is the release of cytokines by immune cells into the circulation. Additionally, pathogen-associated or damage-associated molecular patterns may enter the circulation and affect the functioning of internal organs and the gut microbiota. Furthermore, endocrine communication is the broadest form, encompassing the hypothalamic–pituitary–adrenal axis (HPA). Neural communication primarily depends on the direct anatomical connections made by the vagus nerve or indirect connections through the ENS. Although there is substantial evidence indicating a link between the vagus nerve and microbiome-to-brain signalling, the neuronal networks underlying the MGBA have not yet been fully elucidated. Further research is necessary to elucidate these circuits.

There is a bidirectional relationship between MGBA and neurological disorders. It is well-documented that the gut microbiome has a profound influence on the CNS in the context of health and disease [18,19,20,21,22]. A healthy microbiota is essential for the normal functioning of the brain and the regulation of emotional behaviours. It is therefore crucial to prevent dysbiosis, which refers to an imbalance between the types of organisms present in the gut. This phenomenon is associated with a range of CNS disorders [20]. This review will discuss the bidirectional relationships between the MGBA and neurological disorders. These disorders are divided into developmental disabilities and metabolic disorders, neurodegenerative diseases (NDDs), immune-mediated nervous system diseases, non-communicable neurological disorders, and mental (behavioural) disorders [23]. In this review, our objective is to provide a comprehensive overview of how the field of MGBA has increased understanding of the influence of MGBA on the brain. This is essential to facilitate research into microbial-based interventions and therapeutic strategies for neurological diseases.

## 2. The Bidirectional Relationships between MGBA and Neurological Disorders

**Figure 1:**
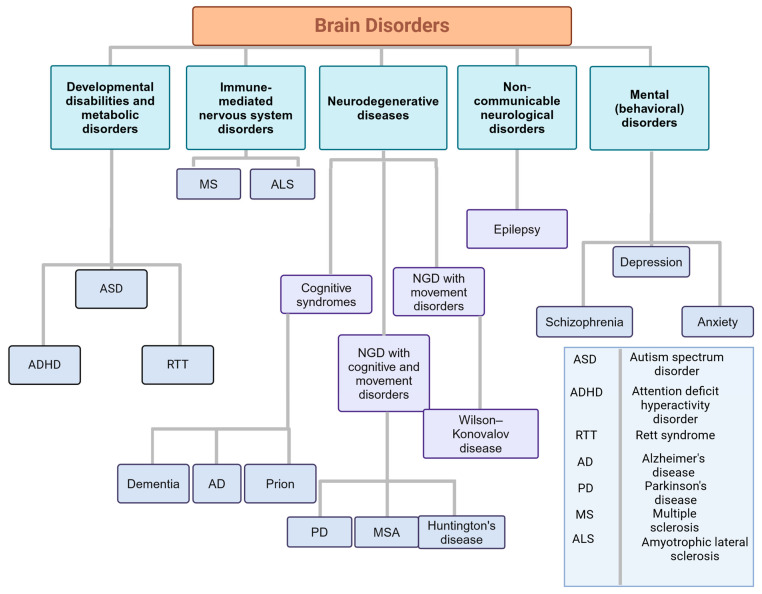
Brain disorder classifications. Neurological disorders are divided in this review into developmental disabilities and metabolic disorders, neurodegenerative disorders (NDDs), immune-mediated nervous system diseases, non-communicable neurological disorders, and mental (behavioural) disorders.

It is well-established that the gut has a profound influence on the CNS in the context of health and disease. A healthy microbiota is essential for normal brain functions and emotional behaviours. Moreover, the CNS controls most aspects of the GI physiology. This chapter will discuss the bidirectional relationships between the MGBA and neurological disorders. These disorders are divided into developmental disabilities and metabolic disorders, NDDs, immune-mediated nervous system diseases, non-communicable neurological disorders, and mental (behavioural) disorders [23] (Figure 1).

### 2.1. The MGBA and Developmental Disabilities and Metabolic Disorders

#### 2.1.1. Rett Syndrome

**Figure 2:**
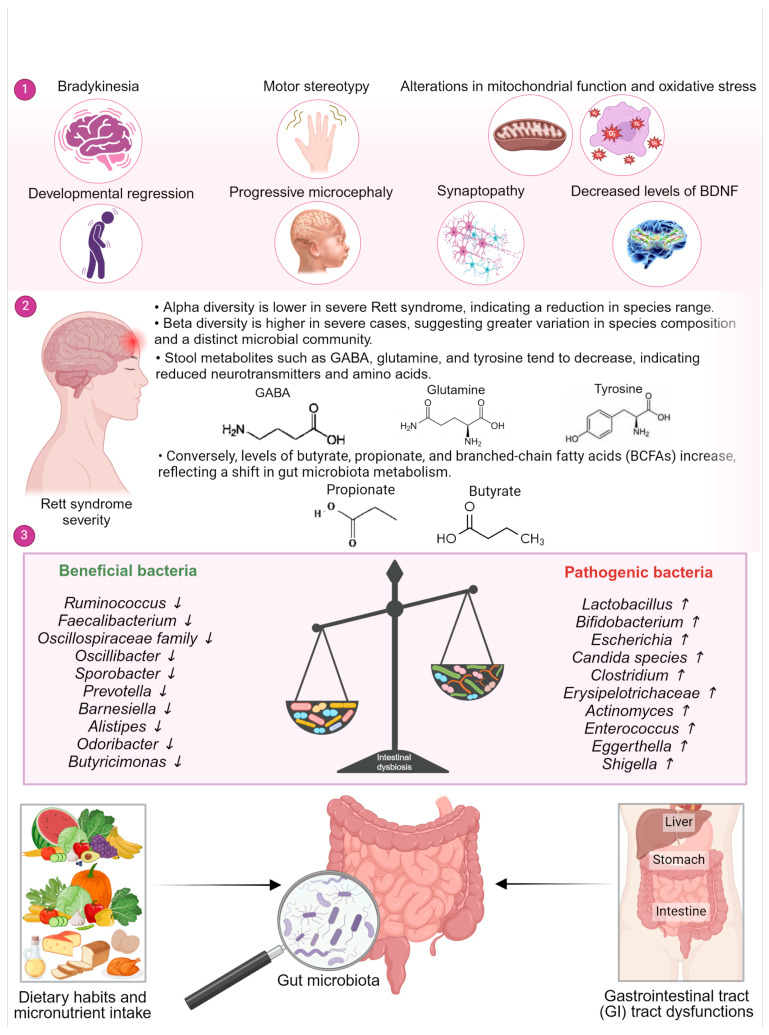
Altered gut microbiota in Rett syndrome. Increased levels of harmful bacteria such as *Clostridium* and a reduction in beneficial bacteria like *Bifidobacterium* and *Lactobacillus* reflect an imbalance in the gut microbial community in patients with Rett syndrome (RTT), indicating an association with gastrointestinal (GI) symptoms such as constipation, bloating, and abdominal pain. Immune modulation, metabolic changes, and the production of neuroactive compounds are mechanisms that may influence the gut–brain axis in RTT patients.

Rett syndrome is a rare X-linked neurodevelopmental disorder, primarily affecting young girls. It is characterized by developmental delay, which manifests as a progressive loss of motor skills and language, accompanied by the development of repetitive hand movements. Neurologically, Rett syndrome is associated with a reduction in brain volume, particularly in the frontal and temporal lobes, as well as abnormalities in the function and structure of synapses [24,25]. The most significant biomarkers include loss-of-function mutations of the X-linked methyl-CpG binding protein 2 (MeCP2) gene, and decreased levels of the brain-derived neurotrophic factor (BDNF) in the cerebrospinal fluid [24,26]. Recent studies have also identified alterations in mitochondrial function and oxidative stress as contributing factors to the disease pathology [27,28]. Several studies have demonstrated that individuals with Rett syndrome exhibit alterations in the composition and richness of their gut microbiome when compared to a control group (Figure 2). These studies have indicated the presence of a potential dysbiosis, which can be defined as an imbalance in microbial diversity relative to the control group. Nevertheless, further research, including larger cohort studies, is necessary to confirm these results. The studies have observed the most notable alterations in the abundance of bacterial taxa within the Firmicutes and *Bacteroidetes phyla*, followed by the *Actinobacteria phylum* in Rett syndrome patients. Notably, a significant increase in potentially pathogenic bacteria has been identified, including taxa belonging to genera such as *Bifidobacterium*, *Clostridium*, *Erysipelotrichaceae*, *Actinomyces*, *Lactobacillus*, *Enterococcus*, *Eggerthella*, *Escherichia*, and *Shigella* in Rett syndrome. The currently available evidence on the genus Bacteroides is inconclusive. The available evidence on this topic is inconclusive, with studies yielding conflicting data [26,29]. In contrast, beneficial bacteria crucial for gut health, including *Ruminococcus* spp., *Faecalibacterium* spp., genera from the *Oscillospiraceae* family (*Oscillibacter*, *Sporobacter*), and several *Bacteroidetes* genera (*Prevotella*, *Barnesiella*, *Alistipes*, *Odoribacter*, *Butyricimonas*), were found to be decreased in Rett syndrome [26,29,30]. Furthermore, the fungal genus Candida was observed to be increased in Rett syndrome subjects compared to the control group [29]. Collectively, these studies indicate that Rett syndrome is associated with dysbiosis in the gut microbiome, characterized by an overabundance of potentially harmful bacteria relative to beneficial species. This microbial imbalance may potentially contribute to the pathophysiology of Rett syndrome. Nevertheless, further investigation is required to determine the relationship between bacterial composition and the severity of Rett syndrome [30]. A list of the bacteria involved in Rett syndrome are listed in (Table 1).

#### 2.1.2. ASD

ASD is a neurological condition that presents challenges in speech, social interaction, and repetitive behaviours. Additionally, individuals with ASD exhibit differences in movement, learning, and attention [32]. Studies on brain imaging have revealed variations in the morphology of the brain, including changes in the amygdala and prefrontal cortex (PFC) size and connectivity. Although studies have indicated potential candidates, such as aberrant levels of specific neurotransmitters and genetic abnormalities linked to synaptic function, the development of biomarkers for ASD is still being investigated [33]. The current research on the gut–brain axis emphasizes the importance of the ENS to play in establishing a vital communication channel known as the gut–brain axis, which links the gut and the CNS via the vagus nerve. This axis affects emotions and behaviour by facilitating communication through immunological reactions, hormones, and neurotransmitters. The human gut microbiome comprises trillions of bacterial cells, which are essential for the proper functioning of the gut–brain axis. The beneficial bacteria such as *Clostridium sporogenes* and *Bifidobacterium infantis* produce metabolites and neurotransmitters that regulate emotions and protect neurological function. However, pathogenic microorganisms like *Clostridium bolteae* and *Clostridium tetani* alter behaviour and neurotransmitter function. They have also been linked to GI problems and an increased severity and risk of ASD. An understanding of these interplays provides valuable insights into potential diagnostic and therapeutic approaches for neurological conditions associated with intestinal dysbiosis. Dysbiosis is associated with a range of disorders, including ASD. This imbalance can lead to disturbances in the host–microbiota equilibrium, with potential consequences for the immune system and the gut barrier. Patients with ASD may have dysbiosis gut microbiota that affects their immune system, causing proinflammatory chemicals to be secreted and changes in gut permeability. Potent proinflammatory endotoxins, such as lipopolysaccharide (LPS), can enter the circulation through this “leaky gut” phenomenon and alter CNS activity, which in turn can impact behaviour, emotions, and neurodevelopment. Research has indicated that individuals with ASD exhibit alterations in certain microbial taxa, including elevated levels of *Proteobacteria*, *Lactobacillus*, *Bacteroides*, and *Clostridium*, accompanied by decreased levels of *Bifidobacterium*, which are good bacteria. These microbiome imbalances underscore the complex link between gut microbiota dysbiosis and ASD pathogenesis, whereby they promote immunological dysregulation, neurotransmitter production, and gut barrier integrity [34].

In patients with ASD, alterations in gut microbiota composition are evident when compared to control groups. Notably, the phylum *Firmicutes* exhibits a decrease, while *Actinobacteria*, *Bacteroidetes*, and *Proteobacteria* show increases. At the family level, *Ruminococcaceae*, *Sutterellaceae*, *Clostridiaceae*, and *Enterobacteriaceae* are increased, whereas *Prevotellaceae* and *Veillonellaceae* decrease. Among genera, *Blautia* experiences a decrease, whereas *Bacteroides*, *Faecalibacterium*, *Ruminococcus*, *Akkermansia*, *Lactobacillus*, *Clostridium*, and *Bifidobacterium* show alterations, with varying increases or decreases. These changes highlight the potential role of gut microbiota dysbiosis in the pathogenesis of ASD.

#### 2.1.3. ADHD

ADHD is a complex brain disorder characterized by persistent problems with attention, hyperactivity, and impulsivity. These challenges affect various behaviours and cognitive repertoires. Some researchers propose that children with ADHD experience a delay in maturation because synaptic pruning lags behind normal development [35,36,37]. The decrease in size in various parts of ADHD brains is frequently linked to a decrease in synaptic density rather than a loss of neurons themselves [38]. The delay in cortical maturation, particularly in the lateral PFC, is evident in children with ADHD. Tasks such as controlling inappropriate responses, directing attention, evaluating rewards, and managing working memory are governed by this region of the brain [36,39]. Conversely, in children diagnosed with ADHD, the motor cortex exhibits a peak in maturation four months ahead of control children. This accelerated development in the motor cortex could lead to the impulsivity often observed in individuals with ADHD [36].

The gut microbiome has been implicated in the pathophysiological mechanisms of ADHD through the MGBA. Alterations in the MGBA contribute to neuroinflammation and oxidative stress, leading to ADHD core symptoms and associated comorbidities such as sleep disturbances. There is some evidence indicating maternal stress and the use of acetaminophen, which is a common pain reliever and fever reducer, may increase the risk of ADHD in offspring during pregnancy [40]. Studies suggest a potential therapeutic role for probiotics in children with ADHD. Probiotic supplementation has shown some promising results in reducing ADHD symptoms and improving cognitive function [41]. Furthermore, the gut microbiome may impact dopaminergic metabolic pathways in individuals with ADHD, suggesting a potential genetic influence on ADHD pathophysiology [42]. The previous study demonstrated that an imbalance in the omega-3/omega-6 PUFA ratio can lead to neuroinflammation and dopaminergic dysfunction, contributing to ADHD symptoms. Pyridoxal phosphate, the active form of vitamin B6, acts as a cofactor for glutamic acid decarboxylase. Glutamic acid decarboxylase converts glutamate into gamma aminobutyric acid (GABA), an inhibitory neurotransmitter, and is involved in the metabolism of tryptophan, which serves as the precursor for serotonin, kynurenic, and xanthurenic acids. Research on tryptophan metabolism revealed that B6 deficiencies are central to the biochemical disturbances seen in ADHD [43].

Bacterial species such as *Bacteroides* spp. and *Clostridiae* spp. are known for their activity in producing short-chain fatty acids (SCFAs). SCFAs can influence neuroinflammation, oxidative stress, and neurogenesis, potentially affecting ADHD symptoms [44]. Prehn-Kristensen and colleagues discovered a higher presence of the *Bacteroidaceae* family in adolescents diagnosed with ADHD [45]. This finding was supported by Wang et al., who identified an increase in certain *Bacteroides* species within the ADHD cohort. However, there were discrepancies in the alpha and beta diversity metrics: Prehn-Kristensen et al. observed reduced alpha diversity and significant variances in beta diversity within the ADHD group, while Wang et al. did not find significant beta diversity differences between groups [46]. These inconsistencies might stem from differences in ADHD medication usage, ethnic composition, and dietary patterns. Probiotics, such as strains of *Lactobacillus* and *Bifidobacterium*, have been investigated for their potential benefits in ADHD. Also, promoting a diet rich in fibre, fruits, vegetables, and omega-3 fatty acids while reducing processed foods and sugars may promote a healthier gut microbiome, which may benefit individuals with ADHD. However, ongoing research shows inconsistent results because of significant methodological differences in factors like sample size, participant selection criteria, the identification of potential confounders, taxonomic composition changes, and potential alterations in synaptic plasticity in ADHD patients, as depicted in (Table 2).

##### The MGBA and NDDs

Since NDDs encompass a wide range of progressive neurological disorders, we want to refer to the clinical classification of NDDs into cognitive and movement disorders. The first group of diseases mainly impacts the hippocampus, entorhinal cortex, limbic system and neocortex, which results in cognitive decline. AD and frontotemporal lobe dementia are typical examples of pathologies from this group. In the second group, the pathophysiological and pathomorphological changes occur in the basal ganglia, thalamus, limbic system, motor cortex, cerebellar nuclei and cortex. Hence, movement system impairment dominates in the clinical presentation, as is seen in WD. The third group of NDs has the structural and functional features of the previous two groups, and it includes MSA, Parkinson’s disease, Huntington’s chorea, and some other pathologies. They present both with cognitive decline and movement disorders.

#### 2.1.4. NDD with Cognitive Syndrome

##### AD

AD, where dementia symptoms insidiously worsen over many years, ranging from mild to severe cognitive decline. While some plaques occur due to aging, large numbers of plaques and neurofibrillary tangles, containing tau and apolipoprotein E, which is a gene that influences the likelihood of developing AD are characteristic features of the neurological disorder AD [51]. Other mechanisms involved in AD include excitotoxicity, gene mutations, amyloidopathy, tauopathy, protein aggregation, oxidative and mitochondrial stress, and neuroinflammation. A parental history of dementia has been associated with a higher risk of developing dementia in both males and females. Currently, nearly 7 million Americans are currently living with Alzheimer’s, and this number is projected to rise to nearly 13 million by 2060 [52]. There is a high degree of bidirectional communication occurs between the GI tract and the CNS via the gut–brain axis. A substantial body of research has demonstrated a robust association between the gut microbiota and the pathogenesis of AD. Consequently, the restoration of a healthy gut microbiota may facilitate the improvement of AD symptoms and progression, such as cognitive function, memory, and visual attention.

1. Gut–brain link to childhood dementia

The potential of gut microbiota therapies for childhood dementia has not yet been fully explored. A distinguishing feature of childhood dementia disorders, such as those characterized by single-gene mutations like AD and MS is their complete penetrance due to genetic alterations, irrespective of dietary patterns, lifestyle, or environmental influences. This genetic predisposition ensures the development of dementia in childhood. In contrast, complex diseases like Alzheimer’s arise from an interplay of genetic susceptibility and environmental factors, such as diet, lifestyle, alcohol consumption, smoking, and pollutants, which may interact with the human genome, inducing the epigenetic modifications of key AD-related genes [53]. While alteration to the gut microbiota may potentially enhance the quality of life for children with these disorders, the therapeutic impact is expected to be less significant compared to diseases influenced by environmental factors. A comprehensive review of the literature on the histopathology of childhood dementia and role of the gut microbiota in neurodegenerative disorders in paediatric populations is lacking. A stronger evidence base is needed to elucidate the mechanisms by which the GI microbiota affects neurodegenerative disorders in children.

2. The bidirectional communication between AD and gut microbiota

**Figure 3:**
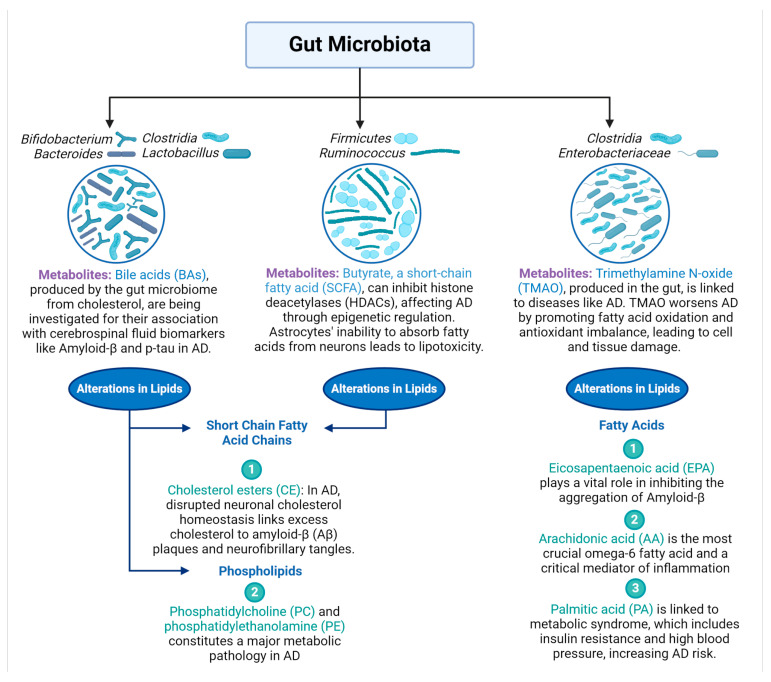
The link between gut microbiota and their metabolites with lipid dysregulation in Alzheimer disease (AD). The involvement of short-chain fatty acids (SCFAs), phospholipids, and other metabolites generated by gut bacteria in the synthesis and degradation of structural and functional lipids in cells might play a role in the progression and deterioration of AD pathology.

**Figure 4:**
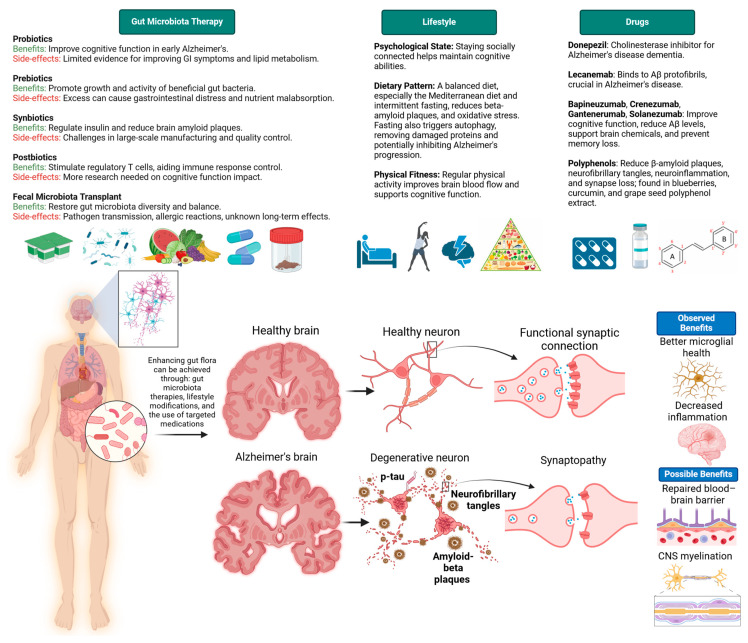
Therapeutic and non-pharmacological interventions employed to enhance cognitive functions in Alzheimer disease (AD). Microbiome-based therapies for AD include probiotics, prebiotics, synbiotics, postbiotics, and faecal material transplantation (FMT). The pathogenic proteins amyloid beta and tau contribute to hypothalamic–pituitary–adrenal (HPA) axis dysregulation, which results in synaptotoxicity and amyloidosis. These interventions aim to modulate the gut microbiota, improve AD symptoms, and modulate plaque-associated microglial functions. However, the beneficial and side effects of these approaches on the central nervous system (CNS), the endocrine system, and the immune system have yet to be fully studied.

Several investigations using animal models have revealed disruptions in the gut microbiota of individuals with AD. For instance, AD mice exhibited increased levels of *Odoribacter* and *Helicobacter*, while *Prevotella* levels decreased [54]. This could indicate a dysbiosis or an imbalance in the gut microbiota. Nevertheless, it is essential to acknowledge that the specific implications of these observed alterations in AD pathology remain poorly understood and necessitate further investigation. A study has demonstrated that *Helicobacter pylori* infection is a risk factor for AD. *H. pylori* can cause an infection in the stomach. Around half of the global population is infected with *H. pylori*, yet only a few genetic variants of this bacterium are pathogenic [55]. The link between gut microbiota and their metabolites with lipid dysregulation in AD is illustrated in Figure 3. Another study showed that mice with AD had higher levels of *Verrucomicrobia* and *Proteobacteria*, while those of *Ruminococcus* and *Butyricicoccus* were lower [55]. Because of the reduced diversity of gut flora in patients with AD, it has been demonstrated to enhance cognitive decline caused by the extracellular clusters of amyloid-beta and intracellular neurofibrillary tangles of tau proteins can be enhanced by faecal microbiota transplantation (FMT) from healthy mouse donors [56]. FMT may positively influence cognitive function by modulating the gut microbiota. This modulation includes reducing the expression of pro-inflammatory cytokines and boosting anti-inflammatory factors, which collectively contribute to improved neural health and cognitive performance. Other therapeutic and non-pharmacological interventions are employed to enhance cognitive functions in AD, as shown in Figure 4. In the study by D’Amato et al. (2020), recipients included twelve male C57BL/6 mice treated with antibiotics, while donors consisted of twelve male C57BL/6 mice, divided into two age groups: young adults (3 months) and older mice (24 months). The FMT was administered via oral gavage for 6 days. Results showed spatial learning difficulties in young mice, a decrease in SCFA-producing bacteria, including *Lachnospiraceae*, *Faecalibaculum*, and *Ruminococcaceae*, and changes in bacteria linked to CNS disorders, such as *Prevotellaceae*, and *Ruminococcaceae*. The observation that microglial cells in the hippocampal fimbria acquired an aging-like phenotype suggests changes in the brain’s immune environment, which can influence synaptic plasticity. Activated microglia can impact synaptic pruning and the overall health of synaptic connections [57]. In another study conducted by Wang et al. (2021), recipients were four 3-month-old specific pathogen-free APP/PS1 mice, while donors were four 16-month-old APP/PS1 mice. The FMT was administered via oral gavage for 1 week. The results showed that pre-antibiotic-treated mice enabled successful gut microbiota engraftment post-transplantation. Astrocyte activation around Aβ plaques was suppressed, unlike microglia, which are involved in Aβ clearance [58]. The suppression of astrocyte activation around Aβ plaques, as opposed to microglia activation, suggests a complex interplay between different cell types in the brain’s response to Aβ pathology. Astrocytes are known to play important roles in synaptic regulation and plasticity, and their altered activation patterns could have downstream effects on synaptic function [58]. This demonstrates that changes in the gut microbiome composition and metabolites may influence signalling pathways involved in synaptic plasticity, such as the production of neurotransmitters or the activation of receptors, and alterations could lead to improvements in the strength and efficiency of synaptic connections, enhancing cognitive function. In the future, the biological mechanisms underlying the improvement of AD through FMT still need to be further dissected.

3. Apolipoprotein E (APOE) influence the composition of the gut microbiota

APOE is a lipid transport protein in the CNS. The genes and functions of APOE in the context of AD have been documented in several research studies [59,60]. APOE facilitates in the transport of cholesterol and lipids between astrocytes and neurons by interacting with the low-density lipoprotein receptor and is involved in receptor-mediated endocytosis of particular ligands [61]. Different APOE genotypes have shown correlations between gut microbiota abundance and AD, using 16S rRNA sequencing, which is the standard for microbial classification and identification, and for quantifying metabolites in faecal samples. Mice with the APOE2 genotype exhibited higher levels of *Ruminococcaceae* and *Prevotellaceae*, bacterial families involved in the production of SCFAs [62]. In mice with the APOE4 genotype and AD, there was an increase in *Lachnospiraceae* and *Deferribacteraceae*, along with a decrease in *Bacteroidaceae* [63]. This was accompanied by reduced concentrations of SCFAs and their precursors, including acetic, propionic, and butyric acid. These findings suggest that APOE genotypes influence the composition of the gut microbiota and the generation of metabolites in AD mice, such as trimethylamine N-oxide, SCFAs, tryptophan metabolites, lipopolysaccharides, and bile acids [62].

##### Non-Alzheimer’s Neurodegeneration and Dementias

The “brain–gut axis” concept was framed after recent findings on the association between NDDs and microbiota [64]. The brain–gut axis BGA is a part of a permanent interaction between the human body and host microbiota. Since the gut hosts the largest human microbiome, the axis is important in maintaining general health. The gut microbiota-to-CNS interaction is bidirectional: the two sides modulate functions of each other through the immune system, hormone, and neurotransmission signalling mechanisms [65,66]. The gut microbiota affects the development of the gut-associated lymphoid system [64]. An animal model of gut dysbiosis showed reduced hippocampal plasticity. In the same rat model, probiotic treatment reduced the hippocampal oxidative stress and apoptosis [67]. Gut dysbiosis disrupts the integrity of the gut barrier and increases its permeability, which forces the passage of metabolites and microbe-associated molecular models from the gut lumen to the mesenteric lymphoid tissue. Normally, this transition is regulated by special immune cells in the lamina propria of the gut, but it is smoothed in neurological diseases [66,68,69]. Many studies have demonstrated the importance of MGBA for maintaining cognitive performance. One of them showed that patients with irritable bowel syndrome exhibit an elevated risk of Alzheimer’s and non-Alzheimer’s dementia due to abnormal bidirectional interaction through MGBA. Byproducts of the dysbiosis in the gut may enter the brain with cytokines released from mucosal immune cells, gut hormones from enteroendocrine cells, and afferent neural pathways. Studies on animals showed that the vagus nerve is the main route for transmitting cytokines, triggering neuroinflammation and neurotoxic substances. Meanwhile, head injury and depression are stronger risk factors for developing both types of dementia [70].

The researchers who studied the metabolites associated with the microbiome in dementia patients found elevated levels of faecal ammonia and reduced levels of lactic acid [71]. This study type focuses on metabolomics, which characterizes small molecules in body fluids, cells, or tissues [72]. Researchers found metabolic changes in the CNS and peripheral blood associated with dementia [73]. The study findings suggest that bacterial species with probiotic properties impact cognitive functioning with the metabolites they synthesize GABA, serotonin, norepinephrine, and acetylcholine [74]. These findings provide an insight into metabolic interactions between the microbiota and host physiology in NDDs. The main roles of the microbiome in brain–gut axis are triggering neuroinflammation and helping the brain fulfil functions with neuroactive and neuroprotective microbial molecules [75]. The understanding of functional interactions of altered microbes with host metabolic pathways is still missing because of the complexity of the microbial community. Furthermore, the major problem of defining the role of brain–gut axis in various diseases is to explain a causal relationship between the microbiome and NDDs rather than finding their correlations only [76]. In the future, genome-scale metabolic models will reveal microbe–microbe and host–microbe interactions to give a comprehensive insight into the role of microbiota on host homeostasis in NDDs.

##### FTLD

FTLD is marked by the progressive atrophy of the frontal and anterior temporal lobes and is associated with the accumulation of Tau protein. The most common form of FTLD is the behavioural variant, which is characterized by deficits in social skills and personality disorders. A recent study suggested that many pathophysiological changes in NDDs—inflammation, immunomodulation, and amyloidogenesis—arise from microbial activity. It demonstrated a prion-like activity of pathogenic proteins in FTLD. The gut microbiota showed specific patterns characteristic for different variants of FTLD: both behavioural and semantic [77]. Few other studies have also examined the connection between gut microbiota and FTLD. Ji et al. investigated the relationships between the abundance of 210 common gut microbiota and five types of dementia [78]. Their findings suggested that *Melainabacteria*, *Rhodospirillaceae*, the *Eubacterium fissicatena* group, *Phascolarctobacterium*, and *Rhodospirillales* might be risk factors for FTLD, whereas *Desulfovibrio* appeared to be a protective factor. More research is needed to understand how these genera might interact with the pathology of FTLD. One possible explanation is that populations of *Rhodospirillaceae* were positively correlated with IL-1, a cytokine linked to cognitive impairment-like behaviours by promoting neuroinflammation and neurodegeneration [79]. In contrast to FTLD, Cammann et al. were the first to report a protective association between the abundance of the *Eubacterium fissicatena* group and AD [80]. This group includes species that metabolize the SCFAs butyrate from dietary carbohydrates [81]. Besides its colonic anti-inflammatory properties, butyrate is essential for maintaining tight junctions that prevent dysbiosis gut permeability [82]. Additional studies are necessary to confirm this microbiome profile, considering that there were only 103 cases of FTLD in the study. Given the clinical and pathological overlap between AD and FTLD, it is reasonable to assume that subsequent studies will confirm altered gut microbiome composition in FTLD.

##### Prion Disease (Creutzfeldt–Jakob Disease)

Creutzfeldt–Jakob disease (CJD), a type of human prion disease, is an uncommon neurological disorder. It can be categorized into sporadic CJD (sCJD), genetic CJD (gCJD), or acquired CJD. CJD results from the accumulation of the misfolded prion protein (PrPSc) in the brain, leading to spongiform changes, neuronal loss, and astrogliosis [83]. Patients with CJD typically present with rapidly progressive dementia, myoclonus, visual disturbances, ataxia, and akinetic mutism. While alterations in gut microbiota are recognized in some NDDs, they have rarely been reported in prion diseases. Recent studies have explored the connection between the gut microbiome and prion diseases, highlighting the role of the gut–brain axis in disease progression through microglial activation, neurotransmitter production, immune modulation, and inflammation regulation [84,85,86,87].

Guo et al. were the first to report gut microbiota changes in humans with prion disease, noting substantial changes of microbiota composition in CJD patients compared to controls [88]. These changes correlated with clinical performance and survival. In prion disease patients, there is a significant reduction in SCFAs due to decreased *Prevotellaceae* [88,89]. SCFAs are crucial for neuroactive functions, including inflammation modulation and neurotransmitter regulation, as well as maintaining gut barrier integrity. The impact of these changes on CJD patients requires further investigation [90,91,92]. Increased levels of *Fusobacteria* have also been observed in CJD patients. Chronic oral *Fusobacteria* infection is linked to AD due to heightened systemic inflammation, which may also play a role in CJD pathology [90,93]. Systemic inflammation increases M cell density in Peyer’s patches, enhancing prion gut absorption and worsening disease progression [94]. Fusobacteria-induced inflammation can accelerate prion disease by affecting microglia cells, which are critical for CNS homeostasis [95]. Nonetheless, future research is warranted to study the pathological role of *Fusobacteria* in prion disease. The gut microbiome’s involvement in neuroinflammation can be triggered by dietary prions, causing dysbiosis and the production of microbial amyloids [84,96]. This process activates the immune system, enhancing microglial and astrocytic activity in the brain, leading to increased neuronal amyloid production and deposition. This proposed interaction between dietary prions, gut dysbiosis, and cerebral amyloidosis highlights potential links between diet and NDDs.

#### 2.1.5. NDD with Movement Disorders

##### WD

WD is a genetic disorder leading to copper accumulation in various organs, including the brain and liver. It is caused by mutations in the ATP7B gene, which impairs copper transport and excretion through the biliary tract. Recent studies have suggested that environmental and dietary factors might influence gene expression in WD [97]. However, the role of intestinal microbiota in WD remains underexplored and warrants further investigation to identify potential microbiota benefits for patients. Geng et al. found that the intestinal flora diversity in WD patients was significantly lower than in healthy controls [98]. Cai et al. reported that the WD group had a significantly lower abundance of Firmicutes, which includes many butyrate-producing bacteria, compared to healthy controls [99]. This reduction could lead to decreased intestinal SCFAs, affecting physiological functions in WD patients. Contrarily, Geng et al. reported higher Firmicutes levels in WD patients compared to controls [98]. The accuracy of these studies may be limited due to their small sample sizes because of WD rarity. Larger studies are needed to establish a clearer microbiota profile in WD patients. Cai et al. also found that WD patients had significantly lower levels of *Actinobacteria* and *Verrucomicrobia*, which are important for intestinal health and glucose homeostasis [99]. The reduction in these probiotics may disrupt physiological functions in WD patients [100,101]. Additionally, the WD group had higher levels of *Proteobacteria* and *Fusobacteria*, which are linked to gut microbiota imbalance and potential pro-inflammatory effects [98,99,102]. WD patients also showed lower levels of *Blautia*, *Ruminococcus*, and *Coprococcus*, which are vital for immune, neurohormonal, and metabolic homeostasis [103,104]. These findings suggest that gut microbiota dysbiosis in WD may be influenced by the host’s metabolic disorders, offering new insights into the disease’s pathogenesis and potential therapeutic targets.

#### 2.1.6. Neurodegeneration with Cognitive and Movement Syndromes

##### MSA

MSA is a progressive, adult-onset neurodegenerative disorder marked by a mix of parkinsonian symptoms, cerebellar ataxia, autonomic dysfunction, and pyramidal signs. The characteristic pathology of MSA includes oligodendroglial cytoplasmic inclusions primarily made of α-synuclein [105]. Both environmental and genetic factors are believed to influence the risk of developing MSA, but the exact causes and mechanisms remain largely unknown. Intestinal inflammation has been implicated in MSA pathogenesis, suggesting a possible role for the gut microbiota in the disease process [106]. Studies examining changes in the gut microbiota of MSA patients are scarce. Engen et al. discovered that American MSA patients had higher levels of *Clostridiaceae* and *Rikenellaceae* but lower levels of *Lachnospiraceae* (including *Ruminococcus*, *Roseburia*, and *Coprococcus*) and *Ruminococcaceae* (including *Faecalibacterium*) in their faecal samples [107]. Tan et al. found that Malaysian ethnic Chinese MSA patients had more *Bacteroides* and fewer *Paraprevotella* in their gut microbiota [108]. Wan et al. also identified distinct microbiota compositions in MSA patients compared to healthy controls [109]. MSA patients had higher levels of genera *Akkermansia* and species *R. hominis*, *A. muciniphila*, and *S. parasanguinis*, and lower levels of genera *Bifidobacterium*, *Blautia,* and *Aggregatibacter*, along with species *M. funiformis*, B. *pseudocatenulatum*, and *G. adiacens*. *Akkermansia* is known for its proinflammatory properties, which include upregulating genes involved in antigen presentation, B and T cell receptor signalling, and complement and coagulation pathways [110]. These proinflammatory effects may result from *Akkermansia’s* disruption of host mucus homeostasis, leading to gut barrier breakdown [111]. Since inflammation plays a significant role in MSA pathogenesis, gut inflammation could increase the risk of MSA. On the other hand, the decreased bacteria, such as *Blautia*, produce butyrate, a SCFA with anti-inflammatory properties [112]. *Bifidobacterium*, another reduced genus, has anti-inflammatory effects and its bioactive compounds enhance epithelial cell barrier resistance, thereby reducing inflammation [113]. These findings suggest that the reduced presence of such beneficial bacteria in MSA patients could contribute to the disease. However, the specific roles of these bacteria in MSA pathogenesis require further investigation.

##### Huntington’s Chorea (HC)

HC is a hereditary NDD caused by the excess trinucleotide repeat expansions in the HTT gene, which results in cognitive deterioration and uncontrolled movements. The symptoms result from dysregulated myelination. Recent studies suggest a significant contribution of the MGBA in the dysregulation [76]. In a HC mice model, gut dysbiosis was first reported in 2018 [114]. Meanwhile, the microbial diversity was higher in the male than female HC mice. Remarkably, the same sexual dimorphism was shown in humans with different NDDs [76]. Researchers have created an animal model of the bacterial artificial chromosome Huntington disease. When compared with pathogen-free mice, the model revealed multiple structural changes in the ultrastructure of the brain. These included a decreased thickness of the myelin sheath in the corpus callosum and a reduction in the number of oligodendrocytes in the PFC [115]. The obtained results indicated the influence of gut microbiota on myelination properties.

##### PD

**Figure 5:**
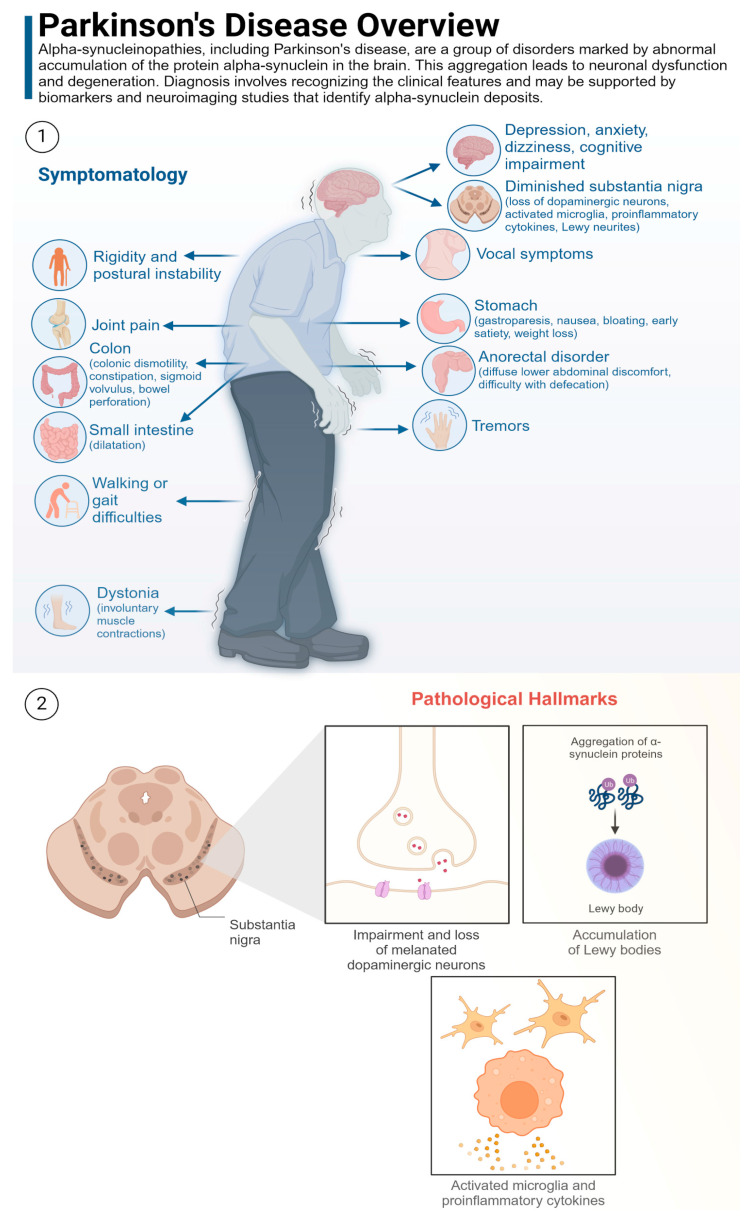
Symptomatology and pathological hallmarks of Parkinson disease (PD). (**1**) Clinical features and symptoms of PD; (**2**) biomarkers and neuroimaging evidence that identifies alpha-synuclein deposits.

PD is a progressive neurodegenerative disorder that is characterized by the degeneration of dopaminergic neurons in the substantia nigra region of the brain. This loss of dopamine-producing cells is associated with the cardinal motor symptoms of Parkinson’s disease, including tremor, rigidity, bradykinesia, and postural instability. In addition to the motor impairments previously discussed, Parkinson’s disease is also associated with a range of non-motor symptoms, including cognitive impairment, mood disorders, and autonomic dysfunction. The primary neuropathological hallmark of Parkinson’s disease is the presence of Lewy bodies, which are abnormal protein aggregates primarily composed of alpha-synuclein [116]. These Lewy bodies are found within the surviving dopaminergic neurons in the substantia nigra (Figure 5) [117]. Furthermore, PD is characterised by the degeneration of other neuronal populations, including noradrenergic neurons in the locus coeruleus, cholinergic neurons in the nucleus basalis of Meynert, and serotonergic neurons in the raphe nuclei [118]. Furthermore, there is a reduction in dopamine transporter binding in the striatum of patients with PD [117]. Other potential biomarkers under investigation include increased levels of alpha-synuclein in the cerebrospinal fluid or blood, and the presence of Lewy-type α-synucleinopathy (LTS) in peripheral tissues like the gut or submandibular gland [119,120,121]. Nevertheless, these biomarkers have not yet been incorporated into routine clinical diagnosis.

A substantial body of research has demonstrated a correlation between PD and alterations in the composition of the gut microbiota. These changes are characterized by an increase in bacteria that may contribute to inflammation and a reduction in beneficial bacteria. Specifically, patients with PD exhibit an increase in phyla such as *Proteobacteria* and *Verrucomicrobiota,* which have been linked to inflammation. Furthermore, families such as *Enterobacteriaceae*, *Peptostreptococcaceae*, *Verrucomicrobiaceae*, *Lachnospiraceae*, and *Ruminococcaceae* are more prevalent in PD cases, while families like *Prevotellaceae* and *Lactobacillaceae* are less abundant [116,122,123,124,125,126,127]. At the genus/species level, *Akkermansia*, a mucin-degrading bacterium that can disrupt the gut barrier, is consistently elevated in individuals with PD [124,125,127,128]. Conversely, beneficial bacteria such as *Blautia*, *Coprococcus*, *Roseburia*, *Faecalibacterium*, *Clostridium* spp., *Bacteroides fragilis*, and *Bifidobacterium* spp. are reduced in individuals with PD [123,127,129]. These findings indicate that gut microbiota dysbiosis in PD is characterized by a shift towards a pro-inflammatory environment, which may contribute to the pathogenesis of the disease. It is therefore evident that further research is necessary to fully comprehend the intricate interplay between gut microbiota and PD and to develop targeted interventions for modulating the gut microbiome to enhance patient outcomes. Further research is necessary to fully comprehend the intricate interplay between the gut microbiota and PD and to develop targeted interventions for modulating the gut microbiome to enhance patient outcomes. A list of bacteria involved in PD are listed in (Table 3).

### 2.2. Immune-Mediated Nervous System Diseases

The immune system is a complex network of cells, tissues, and organs that defend the body from infection. It primarily consists of the innate immune system and the adaptive immune system. The innate immune system has myeloid cells that attack any pathogen that enters the body, including neutrophils, macrophages, and dendritic cells. Pathogens can be consumed by these specialized cells and killed inside the cell [131]. In contrast, the adaptive immune system, consisting of T- and B-lymphocytes, responds to specific antigens. T cells detect pathogens within host cells, forming cellular immunity, while B cells generate antibodies that circulate in bodily fluids, contributing to antibody-mediated immunity [132]. In the gut, innate immunity is triggered by intestinal epithelial cells (IECs) exposed to microbial products. These cells secrete antimicrobial peptides (AMPs), which are crucial mediators of intestinal homeostasis that enable the establishment of an immunological environment permissive to colonization by commensal bacteria [133,134], outlining the gut microbiota’s role in immune defence. The gut microbiota influences T-cell differentiation involved in the adaptive immune system. Gut microbes play a pivotal role in the development of various T-helper cells (Th1, Th2, Th17) and regulatory T cells, which are essential for modulating immune responses [135,136]. SCFAs, such as butyrate, produced by gut bacteria, have been demonstrated to support the formation of regulatory T cells and to help mitigate systemic inflammation. Furthermore, SCFAs have the capacity to reprogram cellular metabolism, thereby promoting the development of regulatory B cells and inhibiting the production of Th17 cells, which is crucial for the management of autoimmune diseases [137]. A substantial body of research indicates that the microbiome exerts a profound influence on Th17 cell differentiation and function. However, the precise mechanisms by which specific bacteria induce intestinal Th17 differentiation remain incompletely elucidated [138]. Consequently, the differentiation of Th17 cells by microbiota colonization is linked across diverse autoimmune diseases, such as MS and amyotrophic lateral sclerosis (ASL), which will be discussed in further detail.

#### 2.2.1. MS

**Figure 6:**
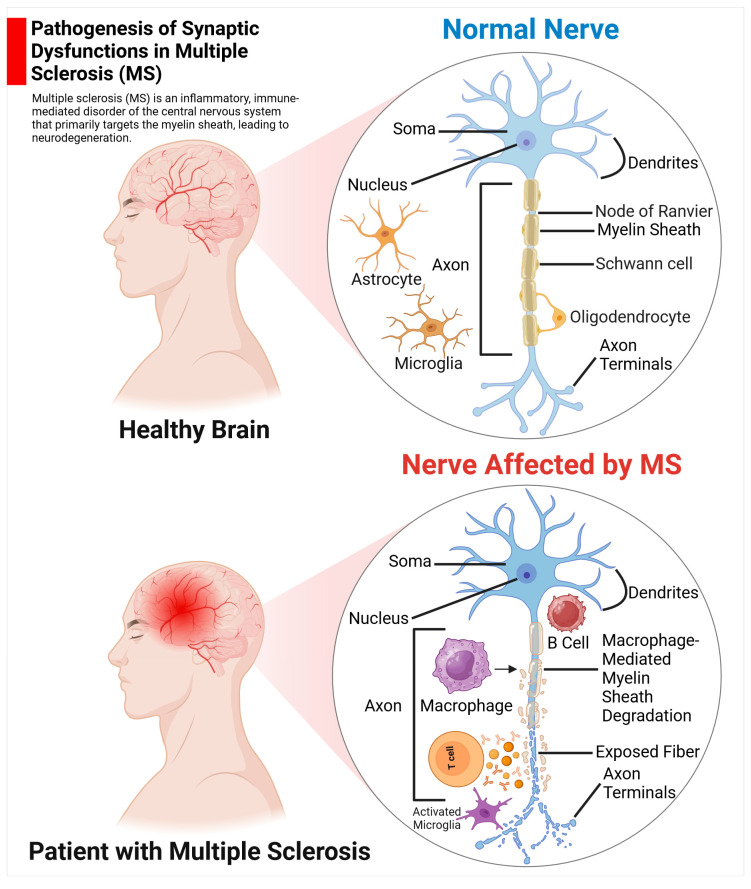
Pathogenesis of synaptic dysfunction in multiple sclerosis (MS). Healthy neurons in MS display normal morphology and function, with intact myelin sheaths that facilitate rapid and efficient signal transmission. In contrast, damaged neurons often exhibit demyelination, where the protective myelin coating is disrupted or lost, leading to impaired signal conduction.

MS is a chronic, inflammatory, demyelinating, and degenerative disease that affects the CNS [139]. This autoimmune condition affects approximately 2.8 million individuals worldwide [140]. Individuals of all age groups may be affected by this condition; however, it is more frequently observed in young adults and is particularly prevalent among females, compared to males. The precise aetiology of the disease remains elusive, although research indicates that genetic and environmental factors may play a role. Its underlying pathophysiology is widely believed to be autoimmune [141]. The primary pathological hallmark of MS is the formation of inflammatory plaques, focal areas of demyelination in the brain and spinal cord as illustrated in Figure 6. This inflammation destroys myelin and oligodendrocytes, leading to neuronal loss [142]. Demyelination is caused by an altered selectivity of the blood–brain barrier (BBB), which allows a wide range of immune cells to infiltrate the CNS lymphocytes that recognize the myelin antigen (CD4+ or CD8+ T cells) cross the BBB and interact with antigen-presenting cells, triggering a cascade of inflammatory events. This process leads to the formation of demyelinating lesions, which are characterized by myelin loss, oligodendrocyte damage, and subsequent neuronal impairment.

MS has traditionally been regarded as a CD4 T-cell-mediated disease. This perspective is largely due to the observation that the major histocompatibility complex (MHC) class II locus constitutes the most significant genetic risk factor for MS [143]. MHC Class II molecules are a subset MHC molecule that are typically found on professional antigen-presenting cells like dendritic cells, macrophages, some endothelial cells, thymic epithelial cells, and B cells. These cells play a critical role in initiating immune responses by presenting antigens to CD4+ T cells, thereby activating them and triggering the adaptive immune response [144]. Even though the aetiology of MS is unknown, one of the key regions associated with MS risk is the major MHC, also known as the human leukocyte antigen. The MHC class II region plays a pivotal role in the immune response. It is involved in interactions with CD4+ (helper) T cells [145]. Moreover, IFN-γ orchestrates a number of protective functions that enhance immune responses. These include the promotion of macrophage activation, the mediation of antiviral and antibacterial immunity, the enhancement of antigen presentation, and the regulation of cellular proliferation and apoptosis [146]. However, the role of IFN-γ has remained paradoxical, with some studies attributing it to a pro-inflammatory and pathogenic function in MS. In particular, CD4+ T helper 1 (Th1) lymphocytes are responsible for secreting interferon-gamma, a cytokine that activates macrophages and stimulates the release of their enzymes. IFN-γ also triggers the production of reactive oxygen and nitrogen intermediates, contributing to tissue damage in the vicinity. Conversely, Th17 cells produce cytokines including IL-17, IL-21, and IL- 22, which are implicated in the inflammatory response and the progression of the disease [147]. Teriflunomide and fingolimod, are two oral disease-modifying drugs used in the treatment of relapsing forms of MS. Fingolimod exerts its effects predominantly on CD4+ T cells, resulting in a reduction in lymphocyte counts in the peripheral blood of MS patients [148]. It is noteworthy that B-cells (which produce antibodies) are reduced, and T helper cells (which control immune cell activity) are also affected, with the exception of natural killer cells, which play a pivotal role in the immune responses against viruses and tumours and are involved in killing the target cells by secreting and delivering perforins and granzymes.

##### The Function of Astrocytes in MS

**Figure 7:**
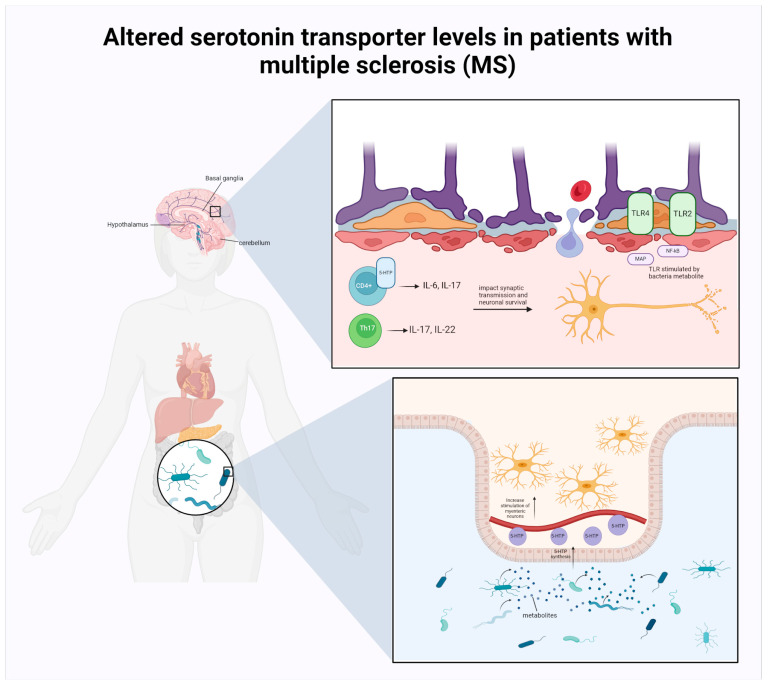
Altered serotonin transporter levels in patients with MS. Tryptophan (TRP) is converted into serotonin (5-HT) through a series of chemical reactions, with the enzyme tryptophan hydroxylase (TPH) playing a key role. Some of the most studied sites of 5-HT action include the gastrointestinal (GI) system, cerebral cortex, and hypothalamus. Activation of the 5-HT1A receptor in clusters of differentiation 4 (CD4+) cells increases interleukin-10 (IL-10) production [164]. Conversely, activation of the 5-HT3 receptor stimulates T cells to produce inflammatory mediators like interleukin-6 (IL-6) and interleukin-7 (IL-17). This demonstrates how serotonin synthesis and its varied roles are intricately connected to the gut microbiota, especially in the context of sickness behaviour.

MS is an inflammatory condition that leads to demyelination and axonal damage in the CNS. The precise aetiology of MS remains elusive. However, emerging evidence suggests that the dysfunction of astrocytes may contribute to the pathogenesis of MS [149]. Astrocytes may play a role in this disease through various mechanisms: (a) by acting as part of the innate immune system; (b) by producing cytotoxic factors, such as reactive oxygen species, nitric oxide, and purinergic metabolites; (c) by hindering remyelination and axonal regeneration through glial scar formation, also known as astrogliosis, which involves changes in cell morphology and molecular expression; and (d) by contributing to axonal mitochondrial dysfunction. Astrocytes may also serve to mitigate the harmful effects of pro-inflammatory factors while offering support and protection to oligodendrocytes and neurons. However, MS represents a poorly understood cellular and molecular mechanism of axonal degeneration and neuronal loss. Furthermore, astrocytes support several activities vital for neuronal function, including (1) playing an active role in the formation and pruning of synapses, which involved eliminating weaker synapses to strengthen existing ones [150]; (2) regulating the extracellular concentrations of ions and neurotransmitters, such as sodium (Na^+^), potassium (K^+^), and calcium (Ca^2+^) ion concentrations [151]; (3) the synthesis of metabolic substrates for neurons, such as glycogen, which serves as an energy reserve, providing glucose when needed during periods of high neuronal activity or low blood glucose levels, while sterols are a critical component of myelin, the protective sheath around axons that ensures efficient signal transmission between neurons from diverse classifications, and lipoproteins, which contribute to neuronal membrane composition, energy production, and signalling processes [152]; (4) the formation and maintenance of the integrity of the BBB, thereby protecting the brain from toxic substances and ions [153]; and (5) the removal of neurotransmitters released by active neurons, such as glutamate, is facilitated through the process of glutamate uptake [154]. This process is essential for maintaining proper neurotransmission and preventing excitotoxicity and glutamate-induced damage in the nervous system. Studies in experimental autoimmune encephalomyelitis, which is the most frequently used model system for studying MS in laboratory animals, especially the brain inflammation, have shown that the loss of their end-feet around small blood vessels are linked to impaired BBB function; high permeability, which can allow harmful immune cells to enter the brain; and CNS inflammation [155]. Astrocytes are responsible for the production of factors that are essential for the establishment and maintenance of endothelial cells, which line the inner surface of blood vessels and lymphatic vessels—they actively contribute to brain function and cognition, orchestrating desirable physiological effects and mitigating adverse effects [156,157,158,159,160,161,162,163]. Inflammatory T cells produce several inflammatory cytokines and chemokines that activate resident glial cells, thus contributing to the breakdown of the BBB, demyelination, and axonal loss [159]. The release of pro-inflammatory cytokines, such as IL-1β, IL- 6, IL-12, IL-17, IL-23, and TNF-α, promotes inflammation and contributes to MS progression [161] (Figure 7). Meanwhile, the release of TGF-β, IL-10, and IL-27 by astrocytes can control the passage of immune cells through the BBB by acting on endothelial cells, tight junctions, and regulating microglial phagocytosis [160]. Astrocytes release molecules that inhibit the differentiation and maturation of oligodendrocyte precursor cells into myelinating oligodendrocytes, thereby impairing remyelination [158]. On the other hand, astrocytes regulate remyelination by the secretion of factors, cholesterol efflux, and the recruitment of peripheral immune cells.

##### Pathological Alterations of Synaptic Structure and Function in MS

Synaptopathy is a neural hallmark of MS pathophysiology This phenomenon is also observed in the early stages of EAE. A multitude of studies investigating synaptic dysfunction in MS have found disturbances in excitatory neurotransmission (mediated by glutamate) and inhibitory neurotransmission (mediated by GABA), which are essential for the proper functioning of the CNS function. Additionally, synapses can be lost due to neuronal injury, also known as synaptic stripping, caused by misfolded proteins and excitotoxic insults. The motor and cognitive functions, including memory consolidation, will be detrimentally affected by synaptopathy, demyelination, and axonal damage. Complements of the immune system, particularly C1q and C3, play critical roles in synaptic refinement and plasticity. C1q is activated during AD progression. C1q is especially associated with the production and deposition of β-amyloid protein (Aβ) and phosphorylated tau in β-amyloid plaques and neurofibrillary tangles (NFTs) in AD, contributing to synapse loss and neurodegeneration in AD [165]. Both C1q and C3 have been recognized as mediators of synapse elimination in the hippocampus of MS patients, indicating a direct connection between inflammation and synaptopathy in MS.

A reduction in inhibitory presynaptic terminals can reduce the amount of neurotransmitter released at each synapse, resulting in less excitation of the postsynaptic neuron. This mechanism has been consistently observed in various brain regions of EAE models. The density of basket and stellate cell inputs to Purkinje cells is significantly diminished in the cerebellum. They fundamentally differ in their dendritic processes. Stellate cells have short dendrites that connect with a small number of Purkinje cell dendrites, whereas basket cells have extensive dendritic processes capable of contacting a much larger number of Purkinje cells. Similarly, in the striatum and primary motor cortex, the number of synaptic terminals identified by the vesicular GABA transporter is reduced. These changes coincide with chronic microglial activation. This shows that synaptopathy happens in different brain regions, such as the cortex, thalamus, amygdala, and hippocampus, in MS patients. The growing recognition of cortical pathological processes and neuronal loss in MS might help elucidate the observed increase in seizure rates among these patients. However, the precise mechanisms by which the myelin-targeting autoimmune reaction leads to abnormal synaptic transmission are not entirely understood. Further investigations are therefore required to elucidate the role of synapse loss in the CA1 and CA3 regions of the hippocampus, which are specifically involved in memory processes, susceptibility to seizures, and neurodegeneration [166].

Studies over the past two decades have illuminated the reciprocal interactions between the CNS and the immune system. During infections, when the peripheral immune response is active, CNS functions are altered, which in turn affects social and psychological activities. The physical symptoms and emotional changes of sick behaviour will be driven by elevated levels of proinflammatory cytokines released by immune cells [167]. This review suggests that there may be greater comorbidity between depression or anxiety, and MS due to the changes in gut microbiota composition which can result in the secretion of stress hormones, including serotonin levels by activating the HPA, and proinflammatory and anti-inflammatory cytokines alterations, affecting synaptic transmission and neuronal survival in MS. Nevertheless, further research is required to elucidate the two-way communication between the intestinal microbiota and the CNS in MS.

##### MS and the Commensal Microbiota

**Figure 8:**
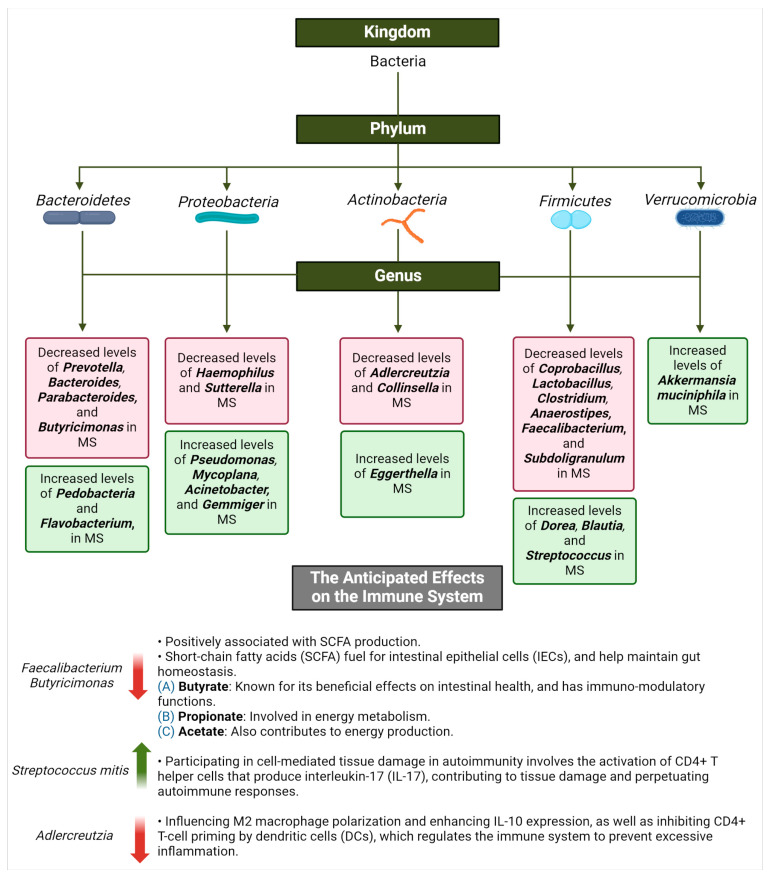
Microorganisms’ alteration in multiple sclerosis (MS). This diagram highlights specific changes in the microbiome associated with the disease and their impacts on the immune system, both beneficial and detrimental.

The alterations in the microbiota composition and diversity may influence the development and progression of MS pathogenesis and other immune-related conditions. Patients with MS have a microbiota with impoverished microbial populations of *Prevotella*, *Bacteroides*, *Parabacteroides*, *Haemophilus*, *Sutterella*, *Adlercreutzia*, *Coprobacillus*, *Lactobacillus*, *Clostridium*, *Anaerostipes*, and *Faecalibacterium* [139]. Other microorganisms are affected in the context of MS, depicting specific changes and their implications in the disease (Figure 8). In fact, commensal microorganisms can promote both regulatory (Th2) and inflammatory responses (Th1 or Th17). This dual effect underscores the intricate role of these microorganisms in regulating immune responses, with the potential to influence the pathogenesis of immune-mediated diseases such as MS [168]. Clostridium difficile is a bacterium that can cause severe enterocolitis, resulting in symptoms like diarrhoea, abdominal pain, and the inflammation of the colon in individuals without underlying disease, and the normal gut microbiota typically controls the growth of *Clostridium difficile*, preventing the development of disease. However, in patients with MS, dysbiosis in the gut microbiota may lead to an overgrowth of *Clostridium difficile*, contributing to intestinal inflammation and potentially exacerbating MS symptoms. *Clostridia* XIVa and IV clusters are specific groups of bacteria within the *Clostridia* class. These bacteria are part of the normal gut microbiota in healthy individuals and play a role in maintaining gut health and immune function. A depletion of species belonging to the *Clostridia* XIVa and IV clusters has been observed in patients with MS, suggesting a potential dysbiosis that may contribute to disease pathogenesis. *Firmicutes* and *Bacteroidetes* are two major phyla of bacteria in the gut microbiota. A balanced ratio of *Firmicutes* and *Bacteroidetes* is associated with gut health and overall well-being in healthy individuals. Alterations in the relative abundance of *Firmicutes*. These alterations could potentially affect neuroinflammation, synaptic function, and neuroplasticity in MS. A pilot study aimed to investigate differences in gut bacteria between patients with MS and healthy controls and evaluate the influence of glatiramer acetate and vitamin D treatment on the microbiota [169]. Vitamin D is a potent immunomodulatory molecule that plays a crucial role in various immune processes within both the innate and adaptive immune systems. It exerts direct effects on T cells, influencing their development and function, and has indirect effects by modulating the activity of other immune cells that interact with T cells. These multifaceted roles underscore the importance of vitamin D in maintaining immune homeostasis and its potential implications in immune-mediated diseases. The study recruited healthy white women with or without relapsing-remitting MS who had vitamin D deficiency. Patients with MS were either untreated or receiving glatiramer acetate, which is an immunomodulator utilized for the treatment and management of MS. The abundance of operational taxonomic units was assessed using a hybridization of 16S rRNA to a DNA microarray. While there was some overlap in gut bacterial communities, several operational taxonomic units, including *Faecalibacterium*, were found to be less abundant in patients with MS compared to healthy controls. The composition of the gut microbiota in glatiramer acetate-treated MS patients differed from that of untreated patients. This was observed in the *Bacteroidaceae*, *Faecalibacterium*, *Ruminococcus*, *Lactobacillaceae*, *Clostridium*, and other *Clostridiales*. Furthermore, untreated MS patients showed an increase in the genera *Akkermansia*, *Faecalibacterium*, and *Coprococcus* after vitamin D supplementation, which was not observed in other groups. The findings of the pilot study suggest that therapeutic interventions in MS, such as glatiramer acetate and vitamin D supplementation, may influence gut microbiota composition, which could potentially play a role in disease management and pathogenesis. Nevertheless, more research is warranted to elucidate the mechanisms underlying these microbiota changes and their impact on MS progression. A number of studies have reported dysbiosis patterns in the gut microbiota of MS patients, including increases in *Akkermansia municipalis* and other bacterial and archaeal taxa [170,171]. Another study deployed 64 untreated MS patients and 68 controls. The bacteria *Acinetobacter calcoaceticus* and *Akkermansia muciniphila* were found to be increased among MS patients, while *Parabacteroides diastonis* was increased among controls. Clinically, in vitro studies have demonstrated that *Acinetobacter calcoaceticus* impairs regulatory T cell (Treg) differentiation and enhances the differentiation of T helper cells (Th1 and Th2). Conversely, *Akkermansia muciniphila* stimulates the proliferation of Th1 cells. In contrast, *Parabacteroides diastonis* produced Tr1, which secretes IL-10. The results suggest that MS-associated changes in microbiota alter T lymphocyte differentiation through multiple mechanisms. More future research on microbial functions in regulating the adaptive autoimmune responses in patients with MS ought to be investigated. Research has also indicated that patients with MS have a higher population of *Enterobacteriaceae* compared to healthy controls [172]. Moreover, a study collected 18 relapsing-remitting MS cases and 17 controls, with a mean age of 13 years. The duration of disease in MS cases was brief, with half of the cases having not been treated with immunomodulatory drugs (IMDs). In comparison to controls, MS cases had an increased abundance of bacteria from the *Desulfovibrionaceae* family (including *Bilophila* and *Desulfovibrio*) and *Christensenellaceae*, along with a decreased abundance of *Lachnospiraceae* and *Ruminococcaceae*. Additionally, microbial genes associated with glutathione metabolism were more prevalent in MS patients, demonstrating that environmental factors may elevate or reduce the colonization of gut microbiota in MS patients [173].

#### 2.2.2. ALS

ALS is a multi-system disorder with progressive atrophy of skeletal muscles, dysphagia, dyspnoea, dysarthria, cognitive dysfunction, and emotional incoherence. The sporadic and familiar cases of ALS are associate with different mutations that trigger an accumulation of transaction response DBA-binding-protein-43 and superoxide dismutase 1 [76]. In ALS, the neurological symptoms come after GU symptoms such as emptying delay and reduced colonic transit timing [174,175]. Meanwhile, the information on the microbial profiles of ALS patients varies across studies. One study showed decreased microbiota diversity in ALS patients [175]. Another study did not justify any significant variation in the gut microbiota as a hallmark of ALS [176]. Yet another publication revealed a profound difference between ALS patients and healthy controls in the microbial profiles [177]. An animal study elucidated the pathophysiological mechanisms of microbiome disbalance in ALS. Those include an increased gut permeability and reduced integrity of membranes constituting the brain–blood and brain–spinal barriers [178]. Despite contradicting findings of previous studies, some research teams are still enthusiastic about the causative and modulating effects of MGBA on ALS pathogenesis [76].

### 2.3. Non-Communicable Neurological Disorders

#### TLE

Both the generation of new neurons in the adult hippocampus (known as adult hippocampal neurogenesis or and the composition of the gut microbiome have been suggested to play a key role in controlling neuroinflammatory mechanisms. The dysregulation of neuroinflammation is a common feature of epilepsy, and interactions between AHN and the gut microbiome may influence inflammatory responses in the brain, contributing to the pathogenesis of TLE [179]. The gut microbiome can influence neurotransmitter production and signalling in the brain. Changes in neurotransmitter levels, such as GABA and glutamate, have been linked to epileptogenesis in the temporal lobe E [180,181]. AHN may also play a role in modulating neurotransmitter systems, potentially affecting neuronal excitability and seizure susceptibility. However, the extent to which gut microbiome–AHN interactions are relevant in the context of epilepsy and seizures remains unclear. Disruptions in adult hippocampal neurogenesis and alterations in the gut microbiome composition could influence the balance between excitation and inhibition in the hippocampus, contributing to hyperexcitability and seizure generation in TLE.

The impaired synaptosomal transport of GABA and glutamate, which is critical for the termination of neurotransmission by the rapid removal of extracellular transmitters has also been implicated in the mechanisms underlying temporal lobe epileptogenesis [182]. Acetylcholine is a neurotransmitter that has been implicated in the pathophysiology of epilepsy, including TLE. Research indicates that cholinergic neurotransmission, involving ACh, is implicated in epileptic activity. However, the biological correlation between ACh and TLE is complex and not fully understood. Additionally, this section discusses the neurotransmitters originating from the dysfunction of the gut–brain axis via the vagus nerve in various neurological and psychiatric disorders. The dysregulation of gut–brain axis signalling has been implicated in conditions such as depression, anxiety disorders, ASD, and NDDs.

Various studies were conducted to investigate the relationship between gut microbiota dysbiosis and TLE in addition to anxiety (Table 4). An analysis was carried out to distinguish differences in the gut microbiota, dissecting them from the broad phylum level down to the more specific genus level [183]. Epilepsy is a persistent neurological condition in which clusters of nerve cells, known as neurons, transmit incorrect signals in the brain, resulting in seizures. Seizure triggers include stress, alcohol consumption, hormonal changes associated with the menstrual cycle, and sleep deprivation. There are two types of seizures: focal seizures and generalized seizures. Temporal lobe TLE is one of the several types of focal seizures. According to Seid et al. (2022), up to 60% of people diagnosed with epilepsy encounter symptoms of anxiety or depression [184]. TLE, recognized as one of the most prevalent forms of epilepsy, affects approximately 65 million people worldwide [185,186]. This condition is characterized by recurrent seizures due to abnormal neuronal activity in the temporal lobes of the brain, resulting in cognitive abnormalities that can adversely influence the overall physical performance in diverse private and public settings. The study hypothesized that various types of epilepsy may lead to distinct prognoses and impacts on the composition of the intestinal flora [183]. According to Munger Clary (2022), anxiety emerges as a prominent psychiatric comorbidity among individuals with epilepsy, showcasing a multifaceted nature encompassing a diverse array of manifestations [187]. These manifestations range from paroxysmal symptoms that have a sudden onset and last only for a short time to anxiety specifically linked to epilepsy itself. Nevertheless, the precise mechanisms that underlie the co-occurrence of epilepsy and anxiety remain unclear at present. The neurotransmitters GABA, norepinephrine, and dopamine neurotransmitters have been identified as key players in the pathogenesis of TLE with anxiety disorder. These neurotransmitters are known to regulate various neuronal functions, including excitability and inhibition in the development and progression of TLEA [188,189]. This study also compares the gut bacteria of individuals with TLEA and patients with TLE but without anxiety disorder. In pathological states like inflammatory bowel disease, T helper 17 cells release pro-inflammatory cytokines that can exacerbate intestinal inflammation. This suggests that Candida albicans may potentially contribute to the development of anxiety in individuals with TLE.

**Figure 9:**
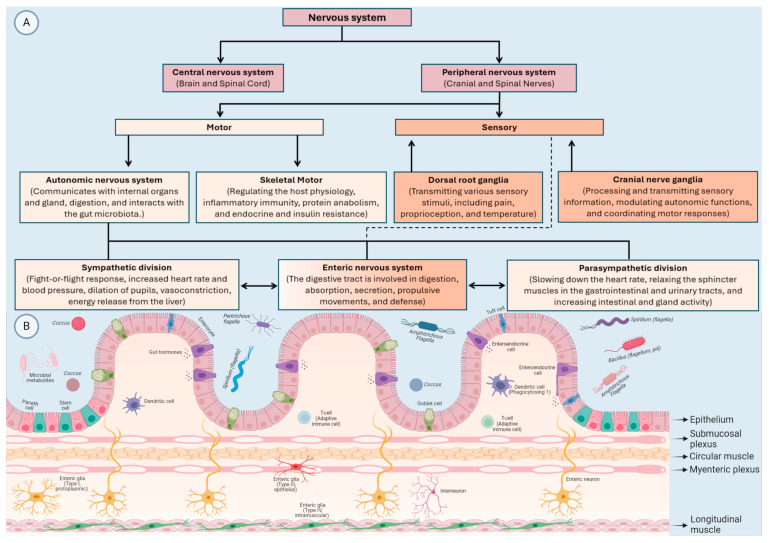
The central nervous system (CNS) and peripheral nervous system (PNS) bidirectionally communicate with the gut microbiota. (**A**) The peripheral nervous system includes the cranial and spinal nerves, in particular, the ganglia nerves that extend beyond the CNS, and the autonomic nervous system, which consists of the sympathetic and parasympathetic branches as demonstrated in. The CNS plasticity includes many cellular and anatomical mechanisms, reflecting synaptic efficacy and synaptic redundancy. The creation of new neurons in the CNS is known as neurogenesis, while synaptogenesis involves the formation of synapses that facilitate neuronal communication. The autonomic nervous system which communicates with internal organs and glands has a flexibility that reflects the integrity of central and peripheral systems, incorporating the adaptation support to environmental demands and thereby serving as a key indicator of neuroplasticity. The digestive tract possesses its distinct nervous system called the ENS. Neurons found in certain nerve clusters transmit sensory information from the body’s outer regions to the CNS. (**B**) The ENS comprises plexuses that consist of neurons. Individual enteric neurons function either as intrinsic afferent, efferent, motor neurons, or interneurons. The myenteric plexus resides between the longitudinal and circular muscle layers. The small intestine alone houses approximately 100 million neurons, making the ENS the largest collection of neurons and glia outside the brain.

The myenteric plexus creates a continuous network spanning from the upper oesophagus to the internal anal sphincter, as illustrated in Figure 9. Submucosal ganglia and fibre bundles form plexuses in the small and large intestines, but not in the stomach and oesophagus. Communication between the ENS and CNS occurs through the vagus and pelvic nerves, as well as sympathetic pathways. In the context of the ENS, neuroplasticity occurs because of inflammation and other perturbations. Changes associated with inflammation-induced neuroplasticity include the increased availability of serotonin in the epithelial cells, the hyperexcitability of intrinsic primary afferent neurons, the facilitation of synaptic activity among enteric neurons, and attenuated purinergic neuromuscular transmission, resulting in dysfunctional motility observed in conditions like colitis, which refers to the inflammation of the large intestine and autoimmune diseases. The hypothesis is that reduced levels of *Lachnospiraceae* and *Ruminococcaceae* may play a role in dysfunctional gut function and increased gut mucosal inflammation in individuals with TLEA [183]. Gut inflammation, whether triggered by abnormal immune responses or gut infections, has been shown to induce neuroplasticity, which includes structural, synaptic, or intrinsic alterations affecting neuronal function. Consequently, neuroplastic changes contribute to irregular secretion, motility, and sensation, ultimately leading to the onset of discomfort and pain [196]. Chen found a higher presence of *Escherichia-Shigella* in individuals with anxiety and noted a positive correlation between the abundance of Escherichia-Shigella and the severity of anxiety symptoms [197]. Additionally, other bacterial groups like Proteobacteria and *Bifidobacterium* showed increased levels not only in patients with anxiety disorders but also in those with TLE with anxiety (TLEA), underscoring the link between gut pathogens and anxiety once again [198]. Moreover, a single-strain probiotic—*L. helveticus R0052*—may decrease seizure susceptibility and this effect can be mediated, at least in part, by increased production of SCFAs [199]. *Escherichia-Shigella* encompasses bacteria known for their pro-inflammatory properties that induce gut inflammation through bacterial structural components such as lipopolysaccharides and bacterial metabolism [200,201].

### 2.4. Mental (Behavioural) Disorders

#### 2.4.1. Depression

Depression is a major mental illness that affects 10% to 15% of the general population [202]. The World Health Organization defines depression as a common mental disorder characterized by recurrent episodes of profound sadness, loss of interest and pleasure in activities, disturbed sleep patterns, and suicidal ideation. In addition, depression has a profound effect on interpersonal relationships and the ability to function effectively in daily life. A significant proportion of the world’s population, particularly in low- and middle-income countries, lacks access to mental health care, despite the availability of effective treatments, due to barriers such as stigma and lack of funding [203]. Depression is characterized by changes in brain morphology, including reduced volume in areas such as the hippocampus and PFC, and abnormal neural circuitry involved in mood regulation. The dysregulation of neurotransmitter systems such as dopamine and serotonin, as well as high levels of stress hormones such as cortisol and inflammatory indicators such as C-reactive protein, are biomarkers associated with depression. These physiological changes underscore the importance of multifaceted approaches to diagnosis and treatment, as they contribute to the complex aetiology and symptoms of depression. Brain imaging studies have identified structural changes in brain areas associated with mood regulation and emotional processing, including the hippocampus, amygdala, and PFC. Potential biomarkers of depression include changes in neuroplasticity-related proteins, such as BDNF, and abnormalities in neurotransmitter levels, including serotonin and dopamine [202]. The gut microbiome plays a central role in the pathogenesis of depression. Alterations in its composition can trigger inflammatory responses that influence behaviour through a variety of pathways, including the HPA. For example, studies have shown that changes in the gut microbiota can lead to an increase in the synthesis of microbial lipopolysaccharides, which trigger inflammatory responses and may be involved in the development of depressive symptoms. In addition, research has shown that the composition and diversity of gut microbes differ between people with depression and those who are not depressed. For example, the gut microbiota of individuals with depression often shows an excess of Firmicutes, Bacteroides, and Actinobacteria, illustrating how dysbiosis can affect mood and behaviour. Furthermore, altered neurobiological states associated with depression, such as elevated neurotransmitter concentrations and inflammation, highlight the function of the gut microbiome in controlling mood and bodily functions. It is therefore essential to understand these interactions if we are to develop targeted strategies to alleviate the symptoms of depression [204].

**Figure 10:**
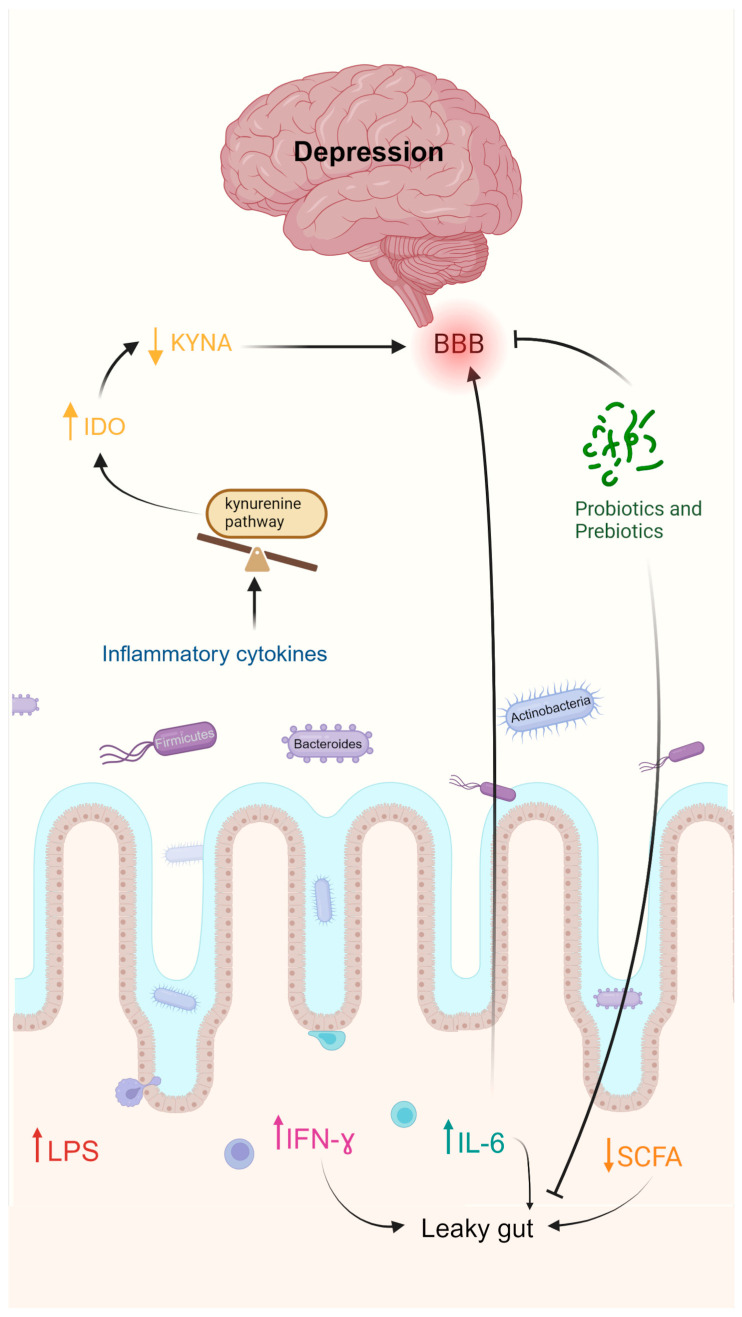
Correlation between the brain and gut microbiome in major depressive disorder (MDD). Stressful circumstances can disrupt the delicate balance of the gut microbiota, resulting elevated levels of proinflammatory cytokines, particularly interleukin-6 (IL-6) and interferon gamma (IFN-γ), and reduced levels of short-chain fatty acids (SCFAs), and weaken the integrity of the gut, facilitating the migration of bacteria (leaky gut). An imbalance in the kynurenine pathway results from increased levels of inflammatory cytokines stimulate the action of indoleamine 2, 3-dioxygenase (IDO), which interferes with the synthesis of protective metabolites such as kynurenic acid (KYNA). As a result, compromising the blood–brain barrier (BBB) increases inflammation in brain tissue and causes astrocyte atrophy and microglial activation. Probiotics and prebiotics have been shown to modify the gut microbiota and improve intestinal barrier function, which in turn indirectly reduces BBB permeability, toxic metabolites from the kynurenine pathway, and inflammatory cytokines. LBS: gut-derived lipopolysaccharides.

There is a robust correlation between the brain and gut microbiome in major depressive disorder (MDD). Stressful circumstances can disrupt the delicate balance of the gut microbiota, resulting in dysbiosis characterized by elevated levels of proinflammatory cytokines, particularly interleukin-6 (IL-6) and interferon gamma, and reduced levels of SCFAs. This inflammatory state can lead to a weakening of the integrity of the gut, facilitating the migration of bacteria (leaky gut). An imbalance in the kynurenine pathway results from increased levels of inflammatory cytokines that stimulate the action of indoleamine 2, 3-dioxygenase, which interferes with the synthesis of protective metabolites such as kynurenic acid. As a result, the toxic metabolites and inflammatory cytokines from this pathway have the potential to compromise the BBB, increasing inflammation in brain tissue and causing astrocyte atrophy and microglial activation. Anxiety and MDD are two examples of mood disorders that may be exacerbated by this chain of events (Figure 10). Conversely, therapies such as probiotics and prebiotics have been shown to modify the gut microbiota and improve intestinal barrier function, which in turn indirectly reduces BBB permeability, toxic metabolites from the kynurenine pathway, and inflammatory cytokines [205].

Taxonomic changes in the gut microbiota have been observed in individuals with depressive disorders compared to controls. These include increased levels of *Firmicutes*, *Actinobacteria*, *Bacteroidetes*, and *Proteobacteria* at the phylum level, decreased levels of *Ruminococcaceae* and increased levels of *Prevotellaceae* at the family level. In addition, specific genera such as *Blautia*, *Faecalibacterium*, and *Coprococcus* show decreases, while *Bacteroides*, *Streptococcus*, *Prevotella*, *Lactobacillus*, *Clostridium*, *Bifidobacterium*, *Eggerthella*, and *Lachnoclostridium* show increases. These findings suggest a potential link between gut microbiota composition and depressive disorders and highlight the need for further research into the role of the gut–brain axis in neurodevelopmental disorders.

Clinical and experimental research suggests that FMT has the potential to reduce symptoms associated with mental illness. Transferring microbiota from a donor with a compromised microbiota to a recipient with a healthy microbiota can cause the recipient to develop symptoms. Conversely, it can alleviate symptoms in recipients who are ill. This suggests that the gut microbiota plays an important role in mental illness. Although FMT from healthy donors regularly reduces symptoms, the duration of relief varies, usually lasting only three to six months. This limitation raises the possibility that FMT may not be a sustainable treatment for mental illness in clinical settings [206].

The MGBA has a bidirectional effect on depression, as evidenced by research showing that introducing microbiota or their metabolites can both cause and treat depression-like symptoms. For FMT, many studies have used faecal samples from depressed humans or mice with depressive-like characteristics. Rats exhibited depressive-like behaviours when exposed to these “depression-related microbiomes” at higher frequencies than controls [207]. On the other hand, it was shown that feeding mice a combination of SCFAs, such as acetate, butyrate, and propionate, could reduce stress-induced depressive-like behaviours [208]. Significant protein changes were found in the serum, prefrontal brain, cecum, and liver of a mouse model of depression induced by FMT from depressed patients, according to a recent proteomics investigation using isobaric tagging for relative and absolute quantitation [209]. These changes in protein profiles were associated with metabolic control and inflammatory immune responses, raising the possibility that the gut–brain axis plays a role in depression. Stronger evidence for the involvement of the MGBA in depression may come from understanding these changes that occur from the gut to the brain or vice versa [210,211,212,213,214].

#### 2.4.2. SCZ

SCZ is a debilitating psychiatric disorder, classified into three primary categories: cognitive symptoms, such as inattention and impaired working memory; negative symptoms, including social withdrawal, reduced motivation, and slowness; and positive symptoms, such as delusions, hallucinations, and disorganized thoughts [215]. The exact cause of SCZ remains unclear, resulting from a combination of genetic, epigenetic, and environmental factors. Numerous neurotransmitters have been linked to the main positive, negative, and cognitive symptoms of SCZ; nonetheless, subcortical dopamine deficiency continues to be the primary cause of psychotic symptoms. Post-mortem studies have shown altered metabolites in the brains of schizophrenic patients, and psychosis in SCZ appears to be mediated by presynaptic dopamine deficiency [216]. However, recent research has indicated that glutamate, GABA, acetylcholine, and serotonin alterations are also involved in the pathology of SCZ [217]. Negative and cognitive symptoms in SCZ are less responsive to antipsychotics and cause significant disability. Importantly, the heterogeneity of antipsychotic responses across individuals, key symptom domains, and biomarker variables require personalized medicine to alleviate the negative, positive, and cognitive aspects of SCZ. Recently, there has been significant focus on the link between human physiology and the microbiome in mental illnesses, especially SCZ.

SCZ gut microbiota differed significantly from those of healthy control subjects and individuals with metabolic syndrome in terms of global composition. Common GI comorbidities in SCZ include irritable bowel syndrome, inflammatory bowel disease, and celiac disease, which is an immune reaction to gluten that damages the small intestine, preventing nutrient absorption [218]. Studies suggest SCZ is associated with gut microbiome disturbances, chronic GI inflammation, and oxidative stress [219]. SCZ gut microbiota were notably enriched in *Flavonifractor plautii*, *Collinsella aerofaciens*, *Bilophila wadsworthia*, and *Sellimonas intestinalis*, while depleted in *Faecalibacterium prausnitzii*, *Ruminococcus lactaris*, *Ruminococcus bicirculans*, and *Veillonella rogosae* [220]. Another study showed changes in the diversity, with associations noted between the microbiome and metabolic and immune pathways, and reported that prebiotics and probiotics can be used as adjunctive strategies in the management of microbiome alterations in patients with SCZ [221]. At the phylum level, Proteobacteria and Firmicutes showed significant variations in SCZ, as did taxa within the class Clostridia, despite one study indicating an overall enrichment of Clostridia in SCZ [219]. Schwarz and collaborators suggest a potential connection between Lactobacilli levels and SCZ progression, correlating with symptom severity [222]. Due to conflicting and limited studies, Further investigation is required to fully understand the microbiome alterations in SCZ, their impact, and potential therapeutic applications.

Moreover, emerging evidence suggests that immune dysregulation, including altered memory T cell function, may contribute to SCZ pathogenesis. A study found an inverse correlation between alpha diversity and a specific subset of memory T cells (CD8+ CD28− CD45RA− cells) in patients with SCZ, highlighting the intricate interplay between the immune system, the microbiome, and the disease process [221]. Dysbiosis may worsen inflammation through increased intestinal permeability. There is a connection between the immune system and the conversion of tryptophan to kynurenate, and the kynurenine pathway plays a role in metabolizing tryptophan, an essential amino acid. The dysregulation of this pathway has been linked to various psychiatric disorders. Kynurenate acts as a broad-spectrum glutamate receptor antagonist, and NMDAR hypofunction is implicated in SCZ [223]. More work is needed to increase the understanding of microbiota–gut–brain axis contributions in SCZ, including increasing sample sizes, excluding potential confounders, accounting for intrinsic variations in microbiome profiles, and ensuring methodological consistency.

## 3. Therapeutics and Their Bidirectional Correlation with the Gut–Brain Axis

### 3.1. Psychotropic Agents

#### 3.1.1. Can the Gut Microbiome Be the New Marker for Safety and Efficacy of Neuro/Psychotropic Drugs?

The evidence is mounting that the gut microbiome affects brain morphology, function, and behaviour, including depression, psychosis, and neurological disorders [18,20,21,224]. For instance, recently, the psychobiotics *Bifidobacterium longum Rosell^®^-175* and *Lactobacillus rhamnosus JB-1* enhances the expression of proteins involved in the activation and maturation of nerve cells, as well as myelination and homeostatic regulation of neurogenesis in mice [225]. It is therefore reasonable to hypothesize that drugs used to treat these conditions may act, at least in part, by modifying the gut microbiome as a potential therapeutic target. Furthermore, some side effects of these medications may be mediated by the effect of these drugs on the gut microbiome. Alternatively, these alterations may serve as a biomarker for the response to various drugs. The following section presents a review of the bidirectional relationship between different CNS drugs and the gut microbiome, based on preclinical and clinical evidence.

##### Psychotropic Agents and Gut Microbiome

1. Antidepressants

The most prescribed antidepressants are selective serotonin reuptake inhibitors. As their class indicates, they primarily act by inhibiting serotonin uptake in presynaptic neurons, thereby increasing its availability [226]. Other classes of antidepressants include serotonin norepinephrine reuptake inhibitors, monoamine oxidase inhibitors, and tricyclic antidepressants, in addition to atypical antidepressants. The evidence of their effect on the gut microbiome can be derived from in vitro, preclinical, and clinical evidence. In vitro, six antidepressants (phenelzine, a monoamine oxidase inhibitor; venlafaxine, a serotonin-norepinephrine reuptake inhibitor (SNRI); (S)-citalopram, a selective serotonin reuptake inhibitor (SSRI); desipramine, a tricyclic antidepressant; and atypical antidepressants (bupropion and aripiprazole) were evaluated for their antimicrobial effect on commensal bacteria. The most notable degree of potentiation was observed in desipramine and aripiprazole. The most abundant phyla in the human gut, namely *Akkermansia muciniphila*, *Bifidobacterium animalis*, and *Bacteroides fragilis*, were found to be most affected by the tested antidepressants [227]. Indeed, the long-term effect of five antidepressants on the gut microbiome and depressive behaviour was evaluated [228]. Two selective serotonin reuptake inhibitors, fluoxetine and escitalopram; two serotonin–norepinephrine reuptake inhibitors, venlafaxine and duloxetine; and desipramine, which acts as a norepinephrine reuptake inhibitor, were administered to a BALB/cOlaHsd mice model (experimental allergic encephalomyelitis resistant). After 21 days of intraperitoneal administration (i.p.), all antidepressants, except for desipramine, were found to reduce alpha diversity richness in faecal microbial communities and increase beta diversity compared to control samples. The 16S RNA sequencing analysis demonstrated that these agents resulted in a reduction in the relative abundance of three genera: *Ruminococcus*, *Adlercreutzia*, and an unidentified genus within the order RF32, class *Alphaproteobacteria*. Behavioural testing demonstrated that duloxetine had a pronounced impact on the tail suspension test, accompanied by a reduction in the relative abundance of *R. flavefaciens* and *A. equolifaciens* species. The former of which mitigated the antidepressant effects of duloxetine, which is attributed to the diminution of mitochondrial oxidative phosphorylation machinery and the impairment of neural plasticity in the medial PFC. Moreover, it has been demonstrated that SSRIs have the effect of increasing the abundance of *Eubacterium ramulus*, while tricyclic antidepressants usage is linked to an enhancement of the populations of *Clostridium leptum*. Duloxetine has been demonstrated to have a remarkable effect of increasing the level of *Eubacterium rectale* by over 100-fold compared to those not taking the medication. Indeed, these specific strains of bacteria are known to produce the anti-inflammatory compound butyrate during their metabolic processes, thus favouring the increase in their levels, which could consequently support the role of antidepressants’ therapeutic action [229]. The sheer volume of data points suggests that antidepressants may exert their antidepressant effects by modulating the composition and functionality of the gut microbiome. A prospective small cohort study revealed that the microbiota composition may predict the success of *levomilacipran* in treating depression in older adults with depression. Specifically, patients exhibiting a proliferation of commensal bacteria, including *Faecalibacterium*, *Roseburia*, and *Agathobacter*, in comparison to *Lachnoclostridium*, demonstrated superior treatment outcomes and improvements in depressive symptoms [230], as measured by the Hamilton Depression Rating Scale. In a study conducted by Shen et al. (2021), the gut microbiome profiles of 30 drug-naive patients with a first episode of depression were compared between those diagnosed with MMD at baseline and after therapy (referred to as the follow-up group) and healthy controls [231]. The variation of alpha and beta diversities was comparable in the follow-up group with the healthy controls and different from the baseline group. The follow-up group exhibited the lowest ratio of Firmicutes/Bacteroidetes, suggesting a “normalizing” effect of escitalopram on the microbiome. Nevertheless, there were still notable differences in the structures and metabolic pathways between the escitalopram-treated group and the control group, which may have reflected a relapse of depressive episodes after four to six weeks of therapy. Similarly, Gao et al. (2023) analysed the faecal samples of MDD patients after 8 weeks of treatment with different SSRIs, including fluoxetine, paroxetine, sertraline, fluvoxamine, citalopram, and escitalopram. This study identified differences in the diversity of the gut microbiome and a higher relative abundance of Blautia, Coprococcus, and Bifidobacterium in treatment-responsive patients compared with treatment-resistant groups. These alterations may be used as markers to predict the response of SSRIs [232]. A larger prospective Chinese study (n = 271) yielded comparable results in patients undergoing SSRIs/SNRIs treatment compared to those undergoing other antidepressant treatment, as well as to patients who had not undergone any antidepressant therapy within the last two weeks of the study commencing. Subsequently, patients in the former group were stratified by the duration of SSRIs/SNRIs use, with those who had been taking them for a longer period exhibiting less microbiota diversity. The relative abundances of seven taxa (*Turicibacter*, *Barnesiella*, *Lachnospiraceae*_ND3007_group, *Romboutia*, *Akkermansia*, *Dialister*, *Romboutia*, and *Fusicatenibacter*) exhibited variability in patients receiving any type of antidepressant. The severity of depression was found to be moderately and inversely associated with Turicibacter in the SSRIs/SNRIs group. The most notable alterations were observed in the pathways of compound biosynthesis and fermentation [233].

Conversely, the gut microbiome can module the side effects of antidepressants. At the level of the gut, the effect of these agents on the enteric serotonin receptors, can lead to known side effects like diarrhoea or constipation, and nausea [234]. These side effects could be attributed to changes in gut microbiome to a different degree between different patients and various SSRIs. Other studies have also showed an ameliorating effect of *R. flavefaciens* on duloxetine-induced constipation [228,235]. To our knowledge, there is no consensus on the effect of antidepressants on the gut microbiome at the preclinical level, but human studies are promising. Larger prospective studies are required to confirm these findings.

2. Antipsychotics

The effect of various second-generation antipsychotics (SGA) on gut microbiome in preclinical studies was well-elaborated [235]. Studies in rodents and human showed that SGAs has antibiotic-like effects and can cause dysbiosis, which can be related to SGAs side effects, such as weight gain, hyperglycaemia, hypertension as well as lipid profile abnormalities. Animal experiments focused on olanzapine and risperidone. In Sprague-Dawley rats, olanzapine caused gender- and dose-dependent changes in gut microbiome. Microbiome isolated from females’ faecal samples exhibited reduced diversity and variation in the abundance of different phyla, including increased *Firmicutes* but decreased *Actinobacteria*, *Proteobacteria*, and *Bacteroidetes*. In males, there was minimal impact on the diversity but similar phyla variation patterns [236]. These changes were correlated with negative effects on metabolism, inflammation, and weight and were mitigated by antibiotic administration [237]. In a GF study of high-fat diet-fed mice, olanzapine had synergistic effect on weight gain and the associated alteration of gut microbiome. There was a decrease in alpha diversity; an increase in the relative abundance of classes *Erysipelotrichi*, *Actinobacteria*, and *Gammaproteobacteria*; and an decrease in abundance of the class *Bacteroidia*. Weight gain was significantly correlated with *Erysipelotrichi* augmentation [238]. Similarly, risperidone brought about changes in wild-type C57BL/6J mice, leading to unfavourable microbiome profiles with correlation to reduced metabolic rate and weight gain in a dose-dependent manner [239,240,241]. Clinically, evidence from human studies on the bidirectional relationship between psychotropics and microbiome was recently reviewed and analysed by Minichiono et al. (2023) [242]. Antipsychotics caused various degrees of alteration in both the alpha and beta diversity of the gut microbiome in cross-sectional studies comparing treated vs. untreated groups, as well as longitudinal studies comparing the same patients before and after the treatment. These studies including second-generation antipsychotics such as olanzapine, risperidone, and quetiapine. In five children with psychosis, the gut microbiome was evaluated 10 months after risperidone treatment and showed an increase relative abundance of *Firmicutes* and *Bacteroidetes*. In a small cohort of young adults, however, there was an elevation in *Bifidobacterium* which was interestingly correlated with weight gain; *Escherichia coli*, after 6-months of risperidone; as well as *Clostridium coccoides* and *Lactobacillus* [243], but an increase in the abundance of *Lachnoclostridium* and a decrease in *Romboutsia*, which was negatively associated with the severity of negative symptoms as assessed by PANSS negative [244]. Bahr et al. (2015) [245], in a cross-sectional manner, showed an increase in alpha diversity in risperidone-treated vs. untreated paediatric patients, and no differences in beta diversity. There was a decrease in the *Bacteroidetes/Firmicutes* ratio (regardless of body mass index), and increased relative abundance of the *Clostridium*, *Collinsella*, *Lactobacillus*, *Ralstonia*, and *Erysipelotrichaceae* family, but decrease in the *Bacteroidetes*, along with the predominance of SCFAs and tryptophan metabolism in the risperidone group. The longitudinal evaluation of 1-month treatment with quetiapine in young adults showed no changes in alpha and beta diversities; however, *Proteobacteria* was increased at the phylum level, as were *Klebsiella*, *Lactobacillus*, *Anaeroglobus*, *Collinsella*, *Paraprevotella*, *Solobacterium*, *Veillonella*, but *Alistipes* was decreased at the genus level. Some of these changes were related to the results seen in the study by Hu et al. (2019) [246], where *Paraprevotella*, *Lachnospira*, and *TM7* increased, while *Acinetobacter*, *Asaccharobacter*, *Eubacterium*, *Lactococcus*, *Lactobacillus*, *Achromobacter*, and *Bifidobacterium* decreased in responders as compared to non-responders. Olanzapine, on the other hand, had neutral effects on gut microbiome and the associated outcomes [247]. A pilot study of 33 SCZ patients evaluated the effect of amisulpride and found that after one month of treatment, there were no significant changes in alpha and beta diversities, but the *Dorea*, *Desulfovibrio*, and *Butyricicoccus* species increased as in *Actinomyces* and Porphyromonas decreased, and there were changes in the butanoate metabolism [248]. Other studied utilized various types of antipsychotics in the intervention group, and thus we cannot elaborate on the effects of specific agents [242].

##### Neurologic Drugs and Gut Microbiome

1. AD pharmacotherapies

AD pharmacotherapy mainly focuses on slowing the progression of disease via the use of agents that enhance acetylcholine concentration in the CNS, which is thought to improve cognitive abilities to various extents [249]. The direct links in currently available AD therapies have not been well studied. Donepezil, a drug used to treat moderate–severe AD, has been shown to modulate the gut microbiome in a mice model of AD (Aβ-injected mice). Mice who received donepezil had significantly more abundance of *Verrucomicrobia* than the control group. There were significances differences in the relative abundance of 12 taxa, including *Blautia* and *Akkermansia* [250].

In the recent years, there has been a focus on immunotherapy that targets the main pathophysiological processes of the disease, namely amyloid beta (Aβ) and tau protein-related plaques [251]. The gut microbiome has been suggested to have bidirectional effect on the immunotherapy. In other words, these agents can induce the alteration of the gut microbiome as part of their mechanism of action; conversely, the gut microbiome can modulate the response to immunotherapy [252].

The gut microbiome of faecal samples of 3xTg-AD mice and wild-type control mice were analysed before and after immunization with the tau antibody 43D on a weekly basis for six weeks. There was a decrease in the phylum *Cyanobacteria* and the order *Turicibacterales* and an increase in the class *Gammaproteobacteria* [252], which is known to affect the integrity of the innate immune system in the gut mucosa. These patterns of *Gammaproteobacteria* and *Turicibacterales* were restored following 43D tau antibody immunization of the 3xTg- AD mouse microbiota.

2. Parkinson’s disease pharmacotherapy

**Figure 11:**
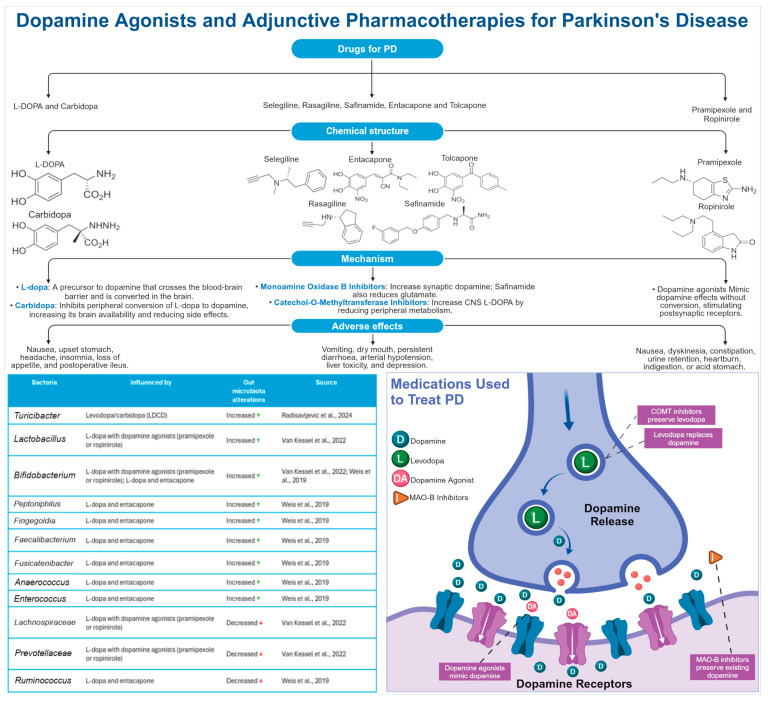
Dopamine agonists and adjunctive pharmacotherapies for Parkinson’s disease (PD). This illustration elucidates how dopamine agonists are potentially adjunctive treatments later in the disease course, along with other approved pharmacologic options for alleviating motor symptoms associated with the disease through the microbiome, contributing to a comprehensive management strategy for the disease. Abbreviations: Levodopa (L-Dopa), catechol-O-methyl-transferase (COMT), Monoamine oxidase-B (MAO-B). Green arrows indicate an increase and red arrows indicate a decrease [254,256,259].

Most therapeutic approaches to treating Parkinson’s disease aim to enhance dopamine levels and alleviate the motor symptoms associated with the disease. Levodopa/carbidopa combination is the cornerstone in the management of Parkinson’s disease. Other therapies can be used as alternatives or add-on therapy, including catechol-o-methyl transferase inhibitors and dopamine agonists (Figure 11) [253]. An in vivo study in the transgenic mice model of PD showed that treatment with LDCD differentially reduced ileal alpha diversity and alleviated nonmotor symptoms of constipation and depression, which occurred alongside a rise ileal rise of *Turicibacter*. Conversely, antibiotic treatment exacerbated constipation, potentially due to lower levels of SCFAs and harm to the integrity of the gut lining. When LDCD and antibiotics were used together, there was a combined effect on behavioural symptoms that correlated with *Turicibacter* abundance in the ileum. This study suggests that, in a mouse model for PD, PD medications and antibiotics may influence non-motor symptoms associated with PD through the gut microbiome [254]. In healthy rats which were exposed to L-dopa in combination of dopamine agonists (pramipexole or ropinirole) for 2 weeks [255], there was a rise in the levels of *Lactobacillus* and *Bifidobacterium* but a decrease in *Lachnospiraceae* and *Prevotellaceae* compared to the vehicle-treated group. Clinically, a study showed that L-dopa and entacapone, a COMT inhibitor, were associated with alteration in the relative abundance of several bacterial genera in 34 PD patients [256]. *Peptoniphilus*, *Finegoldia*, *Faecalibacterium Fusicatenibacter*, *Anaerococcus*, *Bifidobacterium*, and *Enterococcus* were increased and *Ruminococcus* was decreased compared to untreated controls. On the other hand, the gut microbiome has been shown to affect L-dopa’s bioavailability. It has shown that the decarboxylase produced by intestinal *E. faecalis* hastens the conversion of the gut L-dopa into dopamine, and then into m-tyramine via dehydroxylase from *Eggerthella lenta*, which reduces its bioavailability and aggravates its side effects [257,258]. Additionally, *C. sporogenes’* deamination of L-dopa results in 3-(3,4-dihydroxyphenyl) propionic acid, with elevated the faecal concentrations observed in L-dopa-treated PD patients [259].

##### Antiseizure Medications (ASMs)

The alteration of gut microbiome by ASMs is revealed in vitro and preclinical studies. Interestingly, ASMs have been shown to produce detrimental effects in the gut, including in the microbiome. The inhibition of more than ten bacterial strains was recorded for carbamazepine, lamotrigine, and topiramate as well an excipient, propyl paraben, found in ASM syrups. As various artificial sweeteners occurred in the ASM compositions, the bacterial strains in the gut environment were stimulated. The former active ingredients also destroyed HT-29 cells, even though the supernatant of *Bifidobacterium longum* exhibited protective effects against carbamazepine and lamotrigine. *A. muciniphila* or mixed supernatants were able to reduce the drug resistance gene expression in HT-29 cell. This interactions of ASMs and gut epithelial cells could be influenced by the metabolites of the gut’s microbes [260]. On the other hand, a recent preclinical demonstrated that the n5-week administration of topiramate consumption enhanced *Lactobacillus johnsonii* levels in the gut microbiome of C57BL/6J mice. The combined treatment of topiramate and *Lactobacillus johnsonii* reduced the incidence of Pentylenetetrazole-related seizures. This, however, was not the outcome when either of the drugs were administered alone. There were also enhanced levels of butyrate and butyrate producing *Lachnospiraceae* in the intestine because of co-treatment, accompanied with elevated levels of GABA/glutamate rate in the cortex [261].

**Figure 12:**
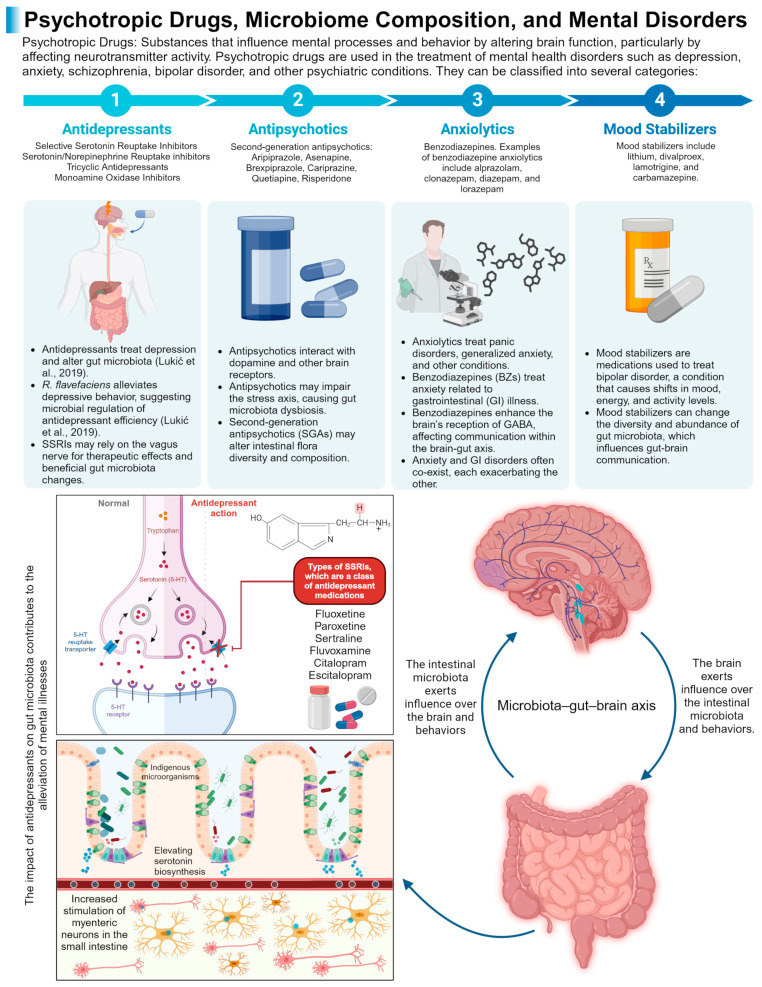
Psychotropic drugs, microbiome composition and mental disorders. Psychotropic drugs work by influencing the levels and activity of neurotransmitters, the chemicals in the brain that transmit signals between nerve cells. The goal of these medications is to correct imbalances in neurotransmitter levels, thereby alleviating symptoms and improving the quality of life for individuals with mental health conditions. This figure explains how psychotropic agents are connected to the gut microbiome, altering its bioavailability. Abbreviations: selective serotonin reuptake inhibitors (SSRIs), gamma-aminobutyric acid (GABA) [228].

In conclusion, the impact of neuro/psychotropic drugs on the gut microbiome and the reciprocal effect is well-documented (Figure 12). The pathways of this interaction may be inferred from the indistinct communication network of the gut–brain axis. However, the detailed molecular mechanisms by which these medications influence the gut microbiome en route to the brain remain to be thoroughly elucidated.

#### 3.1.2. Can Interventions Replenish Gut Microbiome Alter Response to CNS Drugs?

Given the well-established relationship between the gut microbiome and CNS disorders, there has been a suggestion that the alteration of the gut microbiota could be a new therapeutic modality for managing the signs and symptoms of various CNS disorders. These strategies may include the use of prebiotics, probiotics, or FMT (discussed elsewhere in this review). In accordance with the definitions provided by the Food and Agriculture Organization of the United Nations, the World Health Organization, and the International Scientific Association for Probiotics and Prebiotics, probiotics are live microorganisms that provide health benefits to the host when administered in adequate amounts [262]. Species of *Lactobacillus*, *Bifidobacteria*, and *Saccharomyces* are the most used [263], which can be found as additives in various food products, dairy items, and as stand-alone dietary supplements [263]. Probiotics, particularly strains of lactic acid bacteria, are recognized for their ability to alter human gut microbiota by inhibiting the growth of opportunistic bacteria [264]. Hence, using probiotics to stimulate the growth and activity of beneficial strains in the gut is seen as a strategy to manage food-borne enteric pathogens. The health benefits of probiotics extend beyond the gut, as they can exert immunomodulatory, anti-inflammatory, and anticarcinogenic effects [264]. Additionally, there is a category known as prebiotics, which are substrates selectively used by host microorganisms that confer health benefits [265]. While most prebiotics are carbohydrate-based, other compounds like phenolic compounds and conjugated fatty acids also meet prebiotic criteria. Prebiotics are known for their health effects, such as inhibiting pathogens and modulating the immune system [265].

In the context of brain disorders, probiotics exert their mechanisms of action by ameliorating many of the pathogenic processes that are involved in CNS disorders. The positive effect of these “beneficial bacteria/precursors” includes improving the balance of affected neurotransmitters and enhancing the metabolism of lipids and short-chain fatty acid production, in addition to their anti-inflammatory, antioxidative effect [266]. The role of probiotics in ameliorating molecular and behavioural aspect of neuro/psychologic diseases is well studied in animals and human [266]. In the preclinical animal models of AD, the administration of prebiotics and probiotics have been shown to improve memory and decrease Aβ deposition. An 8- to 10-week administration of combined *Lactobacilli* and *bifidobacteria* spp. (1 × 10^10^ CFU/g) increased acetylcholine, ameliorated oxidative stress, inflammation, and improved different aspects of memory in AD models of rats [267,268,269].

A probiotic formulation, SLAB51, which is a formulation consisting of nine live bacterial strains (*Streptococcus thermophilus*, *bifidobacteria* (*Bifidobacterium longum*, *B. breve*, *B. infantis*), *lactobacilli* (*Lactobacillus acidophilus*, *L. plantarum*, *L. paracasei*, *L. delbrueckii* subsp. *bulgaricus*, *L. brevis*), at a dose of 200bn bacteria/Kg/day, has shown to offset brain oxidative-related damages in transgenic AD mice (3xTg-AD) by triggering SIRT1-dependent mechanisms [270]. Human studies in AD patients have shown that after 12-week treatment with probiotic mix (2 × 10^9^ CFU/g of *L. acidophillus*, *L. casei*, *L. fermentum*, and *B. bifidum*) improved sugar and lipid metabolism and had favourable metabolic, anti-inflammatory effects [271], while patients on 1 × 10^10^ of *B. breve* showed improved various aspects of memory including short-term, visuospatial, and delayed memory in geriatric AD patients in a 16-week follow up [272]. Clinically, a large, long-term prospective study showed that a prebiotic intake (fructan), was associated with significantly less risk of developing AD in geriatrics that was not altered by smoking, alcohol intake, gender, race, or APOE genotype.

In Parkinson’s disease, the evidence is still emerging and comes from a few preclinical and clinical studies. A mouse model of PD that received a 10^10^ CFU probiotic mix over 16 weeks displayed less degeneration of dopamine neurons and thus less worsening of motor dysfunctions. PD patients who received high-CFU probiotics had favourable changes in their disease scales and few metabolic profiles. Moreover, a multi-strain probiotic improved non-motor symptoms of PD such as bowel movement and constipation. Sun et al. (2022) conducted a randomized-controlled trial to investigate the effect of add-on probiotic formulation [*Bifidobacterium animalis* subsp. *lactis* Probio-M8 (Probio-M8)] in 42 patients with PD on therapeutic outcomes. After a 3-month follow-up, it was observed that the group receiving Probio-M8 exhibited a significantly higher number of species-level genome bins for *Bifidobacterium animalis*, *Ruminococcaceae*, and *Lachnospira*, but a reduced presence of *Lactobacillus fermentum* and *Klebsiella oxytoca.* Notably, there was a positive association between Lactobacillus fermentum and the scores and disease progression assessment score. Conversely, Klebsiella oxytoca showed a negative correlation with the firmness of the stool. Additionally, when Probio-M8 was used alongside traditional treatments, there was an increase in SGBs associated with the breakdown of tryptophan, the production of GABA, SCFAs, and secondary bile acid production, as well as higher levels of serum acetic acid and dopamine. Interestingly, a prebiotic powder [Bimuno™ galactooligosaccharides (B-GOS^®^)] ameliorated olanzapine-mediated negative metabolic consequences and weight gain in Spraque-Dwaley rats, but no concomitant changes in gut microbiome were observed. Similarly, several types of gut anaerobic bacteria, such as *Clostridium*, *Eubacterium*, and *Bacteroides*, have been shown to possess nitroreductase activity, an enzyme known of its ability to reduction benzodiazepines, including clonazepam. In studies involving human subjects, consuming *L. acidophillus* notably decreased the levels of faecal ß-glucuronidase, nitroreductase, and azoreductase by two to four times. Consequently, combining a probiotic with clonazepam could potentially lessen its toxicity [273].

To date, there is no definitive cure through psychologic/neurologic treatments; therefore, in these settings, pre/probiotics are recommended as adjunctive therapy, which is added to pharmacotherapeutic regimens. However, the effect of pre/probiotics on drug response to CNS disease is still an emerging area of research. How this mechanism is translated into response in these diseases is not well-investigated. Potential indirect pathways can be related to their effects on drug disposition and metabolism; the amelioration of pharmacotherapeutic agent side effects; immune system modulation; the alteration of the levels of neurotransmitters, such as serotonin, GABA, and dopamine, involved in these diseases; immune system modulation; and/or by having direct effects on the brain.

#### 3.1.3. The Effect of Antibiotic-Induced Dysbiosis

There is emerging evidence that suggests a bidirectional relationship between antibiotic-induced dysbiosis and changes in brain functions and structures in animal models and humans through various mechanisms. In mice, the variation in various neurologic functions and structures was evaluated in pups who were exposed to antibiotics in utero [274]. Maternal antibiotics administration is among the most widely used therapeutic approaches in pregnancy. Although published evidence discussed later in this review demonstrates that infants were exposed to antibiotics immediately after birth and while weaning have altered behavioural changes later in life [12], there is a paucity of knowledge regarding the in utero effects of antibiotics on the neuronal function and behaviour of children after birth. The study by Shepilov et al. (2023) aimed to evaluate the impact of MAA at different periods of pregnancy on memory decline and brain structural alterations in young mouse offspring after their first month of life. This study involved pregnant 2–3-month-old C57BL/6J mice. The study consisted of three groups: one control group and two antibiotic-treated groups. The control group (group 1) was provided with sterile drinking water throughout the entire period of gestation, which lasted three weeks. The first antibiotic-treated group (group 2) was exposed to a mixture of amoxicillin and azithromycin in drinking water starting from the second week of pregnancy, with daily administration for seven days. Thereafter, they were provided with only sterile drinking water until birth. The second experimental group (group 3) received the same mixture of antibiotics, but in the third week of pregnancy until delivery. Following delivery, behavioural tests were conducted on the offspring mice during their fifth week of life. These tests were designed to evaluate behavioural changes. The Morris water maze test, a test for spatial learning in rodents [275], and the novel object recognition test, a test commonly used for the investigation of various aspects of learning and memory in mice [276], were employed. The Morris water maze test demonstrated that the administration of antibiotics to pregnant mice in both experimental groups, groups 2 and 3, resulted in a notable reduction in spatial reference memory and learning abilities in their offspring when compared to the offspring of the control group. In contrast, no significant difference in long-term associative memory was observed between the offspring groups in the novel object recognition test. Regarding the structure of the brain and the use of antibiotics, the study employed a histological evaluation of brain samples from the offspring. The researchers employed conventional immunofluorescence and electron microscopy assays, which revealed alterations in hippocampal CA1 pyramidal neurons. These neurons are responsible for processing sensory and motor cues to form a cognitive map encoding spatial, contextual, and emotional information [277]. Additionally, the researchers observed changes in the corpus callosum. The reduction in hippocampal CA1 pyramidal neurons was observed in the groups of mice that were exposed to antibiotics in utero, while the hypomyelination of the corpus callosum was observed in the offspring of treated mice. In addition, both groups of treated mice exhibited a reduction in astrocyte cell surface area and astrocyte territories, respectively, as well as a depletion of neurogenesis in the dentate gyrus and hippocampal synaptic loss. Although this study does not provide evidence that dysbiosis has occurred, it does demonstrate that MAA at different times during pregnancy can pathologically alter cognitive behaviour and brain development in offspring at an early age after weaning [274].

It is well established in the scientific literature that adolescence and early adulthood represent crucial periods in terms of brain development. In a 2015 study, Desbonnet and colleagues assessed the effects of gut dysbiosis on adult cognitive, social, and emotional behaviours in weaned mice. The study used antibiotics to induce gut dysbiosis in the mice. The mice in this study were administered a combination of antibiotics, including ampicillin, vancomycin, ciprofloxacin, imipenem, and metronidazole, beginning at weaning. Both control and antibiotic-treated mice in this study received the same autoclaved pelleted diet. Subsequently, at a later stage of adulthood, between 55 and 80 days postnatal, the researchers assessed the potential neuromodulators of the gut–brain axis, which included tryptophan, monoamines, and neuropeptides, as well as the expression of BDNF. The results of the assessment indicated that depletion of the gut microbiota resulted in reduced anxiety, cognitive deficits, alterations in the dynamics of the tryptophan metabolic pathway, and a significant reduction in BDNF, oxytocin, and vasopressin expression in the adult brain [12]. The alteration in the dynamics of the tryptophan metabolic pathway observed in this study has been demonstrated to affect cognitive performance. Studies have indicated that alterations in the tryptophan metabolic pathway may be associated with specific neurological deficits. These deficits have been observed to be associated with poor cognitive performance and an increased risk of AD and related dementias (ADRDs) [278]. There is a relationship between BDNF and neuronal growth and synaptic plasticity. It has been demonstrated that a reduction in BDNF levels is associated with stress susceptibility, which in turn is linked to a reduction in the synaptic expression of glutamate receptors. This ultimately results in a decrease in excitatory signalling from layer V pyramidal neurons and working memory deficits [279]. In terms of BDNF, it plays a pivotal role in stimulating neuronal growth and synaptic plasticity. Its depletion has been linked to stress susceptibility, which is associated with a reduction in the synaptic expression of glutamate receptors. This ultimately leads to decreased excitatory signalling from layer V pyramidal neurons and working memory deficits [279]. Recent research indicates that oxytocin and vasopressin play a role in cognitive functioning and are involved in the formation of social, working, spatial, and episodic memory [280]. In this study, it was also demonstrated that the severity and longevity of the behavioural abnormalities are associated with the duration of gut dysbiosis during adolescence. The results of the study further elucidate the significant association between gut dysbiosis and brain structure and function.

In the study by Ceylani et al. (2018), 21-day-old male BALB/c mice were administered an antibiotic mixture comprising ampicillin and cefoperazone [281]. The mice were randomly assigned to one of four groups: a control group and three experimental groups that received the antibiotic treatment. The experimental groups were divided based on the orderly or mixed fashion in which they received their treatment. The administration of antibiotics was conducted via drinking water, which each group had free access to. The treatment lasted for a week and was repeated three times, with a one-week interval between each repetition. Following the conclusion of the treatment regimen, at two months of age, behavioural assessments were conducted on the mice. The behavioural tests included the open field test, elevated plus maze test, forced swim test, and novel object recognition test. The open-field test is a commonly employed methodology for the assessment of general locomotor activity and anxiety in rodents [282]. The results of the open-field test demonstrated a significantly reduced locomotor activity in the antibiotic-treated groups in comparison to the control group. The elevated plus maze test is a widely used behavioural test validated to assess anxiety levels in small rodents [283]. The results of this test revealed that most of the treated mice spent a greater amount of time in different areas of the maze than the control group, which confirmed that the treated mice exhibited increased anxiety levels. The forced swim test, a widely utilized procedure for investigating depressive-like behaviours in rodents [284], demonstrated that treated mice exhibited a greater duration of immobility than the control group, thereby substantiating the presence of behavioural despair in the treated mice. Regarding the results obtained within the experimental groups, the ampicillin-treated groups exhibited elevated anxiety levels as evidenced by the open field and elevated pulse maze tests, as well as an increased behavioural despair in the forced swim test. The control group in the novel object recognition test demonstrated a greater propensity to explore novel objects, resulting in lower learning scores for the treated groups. Following the completion of the behavioural tests, blood biochemical markers were evaluated. This included the assessment of BDNF and corticosterone concentrations. The treated mice exhibited lower BDNF levels, which have been associated with changes in affective-like behaviours. Regarding corticosterone levels, no significant difference was observed between the control and experimental groups.

Adult neurogenesis has been linked to hippocampus-dependent cognitive function and is required for memory resolution and proper pattern separation in the dentate gyrus of the hippocampus. Hippocampal neurogenesis may be negatively impacted in various ways, including chronic stress [285] and social isolation [286]. Kempermann investigated the potential association between antibiotic-related gut dysbiosis and its consequences on hippocampal neurogenesis. The study was conducted on adult C57BL/6 mice that were treated with broad-spectrum antibiotics for seven weeks. The antibiotic compounds were administered via drinking water and consisted of ampicillin plus sulbactam, vancomycin, ciprofloxacin, imipenem plus cilastatin, and metronidazole [287]. The researchers employed antibody stains against neuronal progenitor cells and mature neurons to analyse hippocampal sections by immunofluorescence. Their findings indicated that cells in the subgranular zone (SGZ) of the dentate gyrus, the area from which adult neurogenesis originates, were significantly lower in the brains of antibiotic-treated mice.

The impact of dysbiosis on the innate immune system was also examined, given evidence that it serves as an additional link between the brain and gut, in addition to the well-known neuronal association [288]. The evidence for such a connection was established in studies indicating that immune cells play a role in maintaining neurogenesis [289]. Thus, by analysing the infiltrating immune cell populations in single-cell preparations from one brain hemisphere of antibiotic-treated mice, the researchers concluded that the proportions of Ly6Chi monocytes decreased. To further connect the depletion to a decrease in neurogenesis, they employed various strategies to alter monocyte number. One strategy involved the genetic deletion of a chemokine receptor, which deters Ly6Chi monocytes from exiting the bone marrow. The results of the deletion indicated that Ly6Chi monocytes are indeed necessary for neurogenesis. The impact of antibiotic treatment on the gut microbiota of 10-week-old male rats was also evaluated [290]. Following the treatment, the investigators conducted behavioural assessments to evaluate changes in spatial learning and depressive behaviour. The spatial learning assessment employed the Morris water maze, a test widely used to assess spatial learning in rodents [275]. The depressive behaviour assessment utilized the forced swim test, a well-validated method for assessing depressive-like behaviour in rodents [284]. The Morris water maze test demonstrated that mice with depleted gut microbiota in adulthood exhibited deficits in spatial memory. In the forced swim test, the mice exhibited increased visceral sensitivity and a greater display of depressive-like behaviours. The molecular hallmarks of gut–brain communication were also investigated by monitoring CNS serotonin concentration along with changes in the mRNA levels of corticotrophin-releasing hormone receptor 1, glucocorticoid receptor, and BDNF, which is considered a hallmark of altered microbiota–gut–brain axis signalling [290]. CNS serotonin levels modulate higher brain functions, including cognition and emotional behaviour. A deficiency of this neurotransmitter is linked with numerous psychiatric disorders [291]. CRH is the principal regulator of the HPA axis and is critical for the adaptation of the organism to environmental changes. A disruption of the normal HPA axis represents a significant risk factor for neuropsychiatric disorders, with the decreased expression of the glucocorticoid receptor having been documented in numerous cases [292]. Regarding BDNF, the depletion of which was previously highlighted, it is involved in numerous activities that are modulated by the HPA axis. The analysis of behavioural investigations and molecular hallmarks indicates that alterations in the gut microbiota of adult mice may still result in the disruption of cognitive functions. It has been demonstrated that antibiotic treatment can result in alterations to brain structure and function, irrespective of the age at which the treatment is initiated. A review of the literature revealed that studies conducted on mice at different ages have demonstrated that antibiotic treatments do indeed cause changes to the brain’s structure and function. These changes have been shown to manifest as alterations in behaviour and the development of neural diseases.

## 4. Other Factors Influencing MGBA

### 4.1. Mode of Delivery

The gut–brain axis may be affected by alterations in microbial colonization that occur during vaginal birth and caesarean section (C-sections). The gut–brain axis is a bidirectional communication system between the gut and the brain that involves multiple channels, including neurological, immunological, endocrine, and microbial interactions. The gut–brain axis is developed and maintained in large part by microbes in the gut. They produce a plethora of metabolites that can influence the communication between the gut and the brain, including neurotransmitters, SCFAs, and neuroactive compounds.

The mode of delivery has been demonstrated to play an important role in determining the initial composition of the gut microbiota in newborns. This has been shown to significantly influence the development of the immune system and long-term health. In humans, vaginal delivery and C-section are the two primary methods of childbirth, each of which results in different microbial exposures for the neonate. Following a vaginal birth, the newborn is exposed to the maternal vaginal microbiota, which facilitates the colonization of the infant’s gut with bacteria belonging to the *Bifidobacteriaceae*, *Bacteroidaceae*, *Enterobacteriaceae*, and *Streptococcaceae* families [293]. This early exposure to diverse microbial species is believed to be important for the establishment of a healthy gut microbiome and the subsequent development of the gut–brain axis. The primary bacterial phyla introduced during vaginal birth include *Firmicutes*, *Bacteroidetes*, *Actinobacteria*, and *Proteobacteria* [294]. Among these, the phyla Firmicutes and Bacteroidetes are of particular significance. The Firmicutes phylum encompasses beneficial genera such as *Lactobacillus* and *Clostridium*. *Lactobacillus* species, which are prevalent in the maternal vaginal microbiota, play a pivotal role in infant gut health by producing lactic acid, which inhibits the growth of pathogenic bacteria [295]. *Bacteroidetes*, with genera such as *Bacteroides*, plays a crucial role in the breakdown of complex molecules and the production of SCFAs, which are essential for gut health [296]. *Actinobacteria*, including *Bifidobacterium* species such as *Bifidobacterium longum* and *Bifidobacterium breve*, are essential for the digestion of human milk oligosaccharides and the promotion of a healthy gut environment [297]. In contrast, the infant does not come into contact with the maternal vaginal microbiota during a C-section delivery, as the birth canal is bypassed. Rather, the hospital environment, the mother’s skin microbiota, and healthcare professionals all have an impact on the initial gut colonization of the infant. These microorganisms include members of the *Staphylococcaceae*, *Corynebacteriaceae*, *Propionibacteriaceae*, and *Clostridiaceae*. It is possible that the gut microbiome may become less optimal and diversified because of this altered microbial colonization. It is possible that long-term consequences may arise on the gut–brain axis due to the altered gut flora of infants born via C-section. It has been postulated that the dysbiosis observed in these infants may result in alterations to the synthesis of immunological molecules, metabolites, and neurotransmitters, which could potentially impact the growth and functionality of the brain [224]. Furthermore, C-sections prevent the vertical transfer of vaginal microbiota, resulting in microbial colonization from skin and environmental microorganisms. The *Firmicutes* phylum shifts towards bacteria associated with the skin, such as *Staphylococcus*. While some species of *Staphylococcus* are harmless, others, such as *Staphylococcus aureus*, can be pathogenic. *Actinobacteria*, such as *Corynebacterium* and *Propionibacterium*, which are common skin bacteria, become more prominent [298]. Furthermore, *Proteobacteria*, which are more prevalent in infants delivered by C-section, include genera such as *Escherichia* and *Klebsiella*, which are often associated with hospital environments and potential opportunistic pathogens [299]. This altered microbial composition in C-section infants is associated with an increased risk of developing conditions such as allergies, asthma, and other immune-related disorders [229].

**Figure 13:**
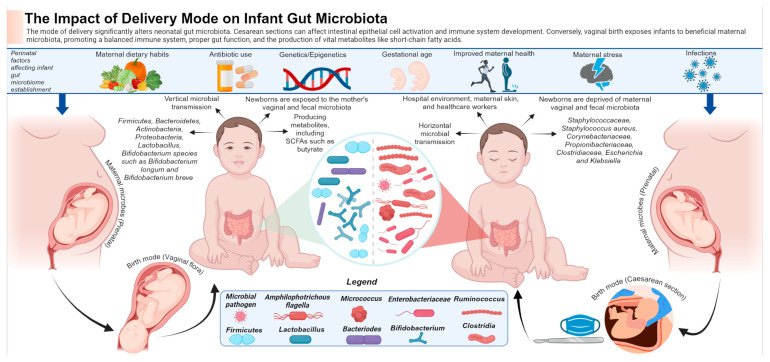
The impact of delivery mode on infant gut microbiota. The mode of delivery significantly alters neonatal gut microbiota. C-sections can affect intestinal epithelial cell activation and immune system development. Conversely, vaginal birth exposes infants to beneficial maternal microbiota, promoting a balanced immune system, gut function, and short-chain fatty acids (SCFAs).

A disruption in the construction and maturation of the gut microbiota, particularly during preterm birth via C-section, has been linked to neurodevelopmental problems and neuroinflammation. Delivery by C-section, particularly in cases of preterm birth, may result in brain abnormalities referred to as “encephalopathy of prematurity”. Preterm birth disrupts the crucial neurodevelopmental program that occurs in the final trimester of pregnancy, causing grey and white matter changes. It is evident that these brain abnormalities have a profound impact on neurobehavioral and cognitive functions. Additionally, it has been established that extreme preterm births, frequently associated with C-sections, are linked to an elevated risk of neurodevelopmental disorders such as ASD. The results of studies conducted on rat models are consistent with those observed in humans, indicating that the method of delivery affects the composition of the gut microbiota (Figure 13). Vaginally delivered rat pups are exposed to the maternal vaginal and faecal microbiota, resulting in an initial colonization of their gut that mirrors that seen in human infants, with a predominance of *Firmicutes* and *Bacteroidetes* [300]. Genera such as *Lactobacillus* and *Bacteroides* are commonly found in the gut microbiota of vaginally delivered rat pups [301]. In contrast, the composition of the gut microbiota in rat pups delivered by C-section differs significantly from that of vaginally delivered rats. The absence of exposure to the maternal vaginal microbiota results in an initial colonization dominated by skin and environmental microbes. The *Firmicutes* phylum in C-section pups includes genera such as *Staphylococcus* and *Corynebacterium*, which are more commonly found on the skin. Additionally, *Proteobacteria*, including potentially pathogenic genera such as *Escherichia*, are also more prevalent. These differences in microbial colonization are associated with increased inflammatory responses and altered immune function in C-section delivered rat pups, indicating potential long-term health implications [302]. Mouse models have also been extensively utilized to investigate the impact of delivery methods on gut microbiota composition, corroborating the observations made in humans and rats. Vaginally delivered mouse pups are exposed to the maternal vaginal and faecal microbiota, resulting in a gut microbiota composition dominated by beneficial bacteria. The most prevalent bacterial phyla are *Firmicutes* and *Bacteroidetes*, with genera such as *Lactobacillus* and *Bacteroides* playing a pivotal role in the digestion of nutrients and the development of the immune system [303]. In contrast, the microbiota composition of C-section delivered mouse pups differs from that of vaginally delivered mice. The initial colonization of the gut microbiota in these pups is influenced by microbes from the skin and the environment, resulting in a less diverse microbiota. The most prevalent bacterial phyla are *Firmicutes* and *Proteobacteria*, with genera such as *Staphylococcus* and *Corynebacterium* being more common. This altered microbiota composition is associated with increased susceptibility to infections and altered metabolic profiles, underscoring the significance of early microbial exposure in influencing long-term health outcomes [302].

The mode of delivery, specifically C-section, has been associated with alterations in inflammatory markers. Several studies have demonstrated that infants delivered by C-section exhibit elevated levels of inflammatory markers in comparison to those born vaginally. One of the most extensively studied inflammatory markers is C-reactive protein. C-reactive protein is a marker of systemic inflammation, and its levels have been found to be higher in infants born by C-section compared to those born vaginally. This suggests that C-section delivery may result in a more pronounced inflammatory response in newborns. In addition to CRP, other inflammatory markers, including interleukin-6 (IL-6) and tumour necrosis factor-alpha, have also been found to be elevated in infants delivered by C-section. These markers are involved in the regulation of the immune response. The elevated levels of these markers may indicate an increased inflammatory state in infants born via C-section. The precise mechanisms underlying these alterations in inflammatory markers in infants born via C-section remain unclear. However, it has been postulated that the differences in microbial colonization between C-section and vaginal delivery may play a role. The initial microbiota of infants born via C-section differs from that of infants born vaginally. This altered microbiota composition may contribute to the increased inflammation observed.

It is crucial to comprehend these distinctions to devise effective strategies to mitigate the potential adverse effects of C-section delivery. Interventions such as the use of probiotics or microbiota transplantation may help restore a more beneficial gut microbiota in infants delivered by C-section, thereby supporting their immune development and reducing the risk of long-term health issues. The mode of delivery thus not only influences immediate microbial colonization but also sets the stage for the infant’s future health trajectory, underscoring the importance of microbial exposure in early life.

The mode of delivery has a profound effect on the neonatal microbiome, which in turn exerts a significant influence on brain development and function through a number of different mechanisms. During vaginal delivery, newborns are exposed to the mother’s vaginal and faecal microbiota, which plays a crucial role in the initial colonization of the infant’s gut. These bacteria are known to produce a number of important metabolites, including SCFAs such as butyrate, which have anti-inflammatory properties and support the integrity of the gut barrier [304]. The gut microbiome also affects the production of neurotransmitters such as serotonin and GABA, which are critical for brain development and function [305]. Conversely, infants delivered via C-section are deprived of this crucial exposure to maternal vaginal and faecal microbiota, and instead become colonised by microbes from the hospital environment, maternal skin, and healthcare workers.

The impact of delivery method on brain structure and function has been the subject of several studies. For instance, infants born via C-section have been demonstrated to exhibit distinct cerebral morphology in comparison to those born vaginally [306]. MRI studies have indicated that C-section delivered infants may have altered white matter integrity and cortical thickness, which are critical for cognitive and motor functions [307]. These structural changes in the brain are believed to be linked to the altered inflammatory environment and microbial colonization associated with C-section delivery. Moreover, the development of the immune system is intimately connected to the gut microbiome. The absence of beneficial microbial exposure in infants delivered via C-section can result in a delayed maturation of the immune system. This can result in an increased susceptibility to infections and a higher risk of developing autoimmune and inflammatory conditions later in life. Furthermore, the chronic low-grade inflammation associated with dysbiosis can also influence brain development and function, potentially leading to neurodevelopmental disorders such as ASD and ADHD [308]. Studies have shown that children born via C-section are at a higher risk of developing these disorders, which are characterized by alterations in brain function and behaviour [309]. In conclusion, the mode of delivery has a profound impact on neonatal microbiome composition, systemic inflammation, and brain development (Figure 13). Vaginal delivery is associated with the establishment of a beneficial microbial colonization that is conducive to the healthy development of the brain and maturation of the immune system. Conversely, C-section delivery is associated with dysbiosis, increased inflammation, and alterations in brain structure and function. These findings underscore the necessity of contemplating the long-term health consequences of delivery methods and illustrate the potential advantages of interventions designed to restore a healthy gut microbiome in infants delivered via C-section.

### 4.2. Exercise

**Figure 14:**
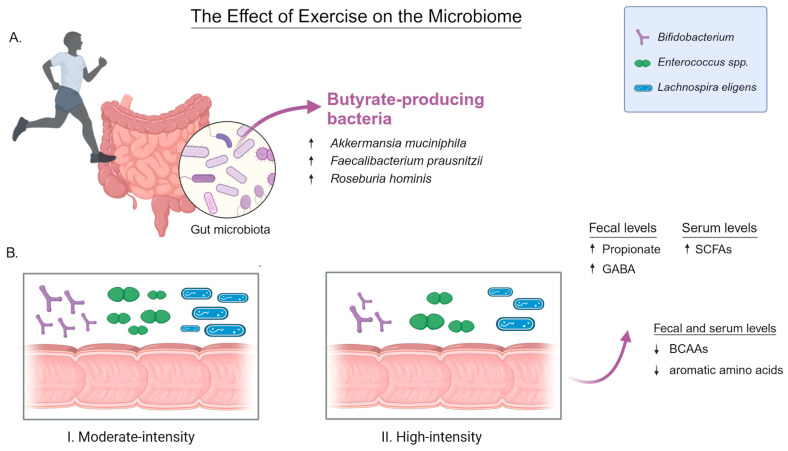
The effect of exercise on the microbiome. (**A**) Individuals who engage in at least three hours of exercise per week exhibited elevated levels of butyrate producing bacteria, including *Akkermansia muciniphila*, *Faecalibacterium prausnitzii*, and *Roseburia hominis*. (**B**) The comparison between moderate and high intensity exercise. The abundance of Bifidobacterium and butyrate-producing bacteria, including *Lachnospira eligens* and *Enterococcus* spp., was greater in individuals who participated in lower intensity exercises. Additionally, obese and overweight males who practiced high intensity exercise presented with reduction in faecal and serum levels of branched-chain amino acids and aromatic amino acids. They also had increased faecal propionate, gamma-aminobutyric acid (GABA), and short-chain fatty acids (SCFAs). Abbreviation: branched-chain amino acids (BCAAs).

The gut microbiome is home to a diverse microbial community. One of the major functions of the gut microbiome is to maintain barrier function and homeostasis. However, several factors can influence its quantitative and qualitative composition. These alterations can have a profound impact on the health of the host [310]. In recent years, numerous researchers have reported the potential positive effects of exercise on gut microbiota (Figure 14). Early indications regarding the potential role of exercise in gut microbiome composition were reported by animal studies and human cross-sectional studies [311]. For instance, Bressa et al. demonstrated that individuals who engaged in at least three hours of exercise per week exhibited elevated levels of butyrate-producing bacteria, including *Akkermansia muciniphila*, *Faecalibacterium prausnitzii,* and *Roseburia hominis* [312]. Similarly, Clarke et al. reported increased gut microbiota diversity in rugby players compared to the control group [313]. Barton et al. also shared similar observations in their study among athletes compared to sedentary controls [314]. Their findings showed that athletes experienced relative increases in certain pathways, such as amino acid and antibiotic biosynthesis, as well as carbohydrate metabolism. Moreover, an increase in faecal metabolite levels, including microbial-produced SCFAs such as acetate, propionate, and butyrate, was observed [314]. However, these studies are limited by their cross-sectional design, which constrains the ability to control diet and other covariates that affect gut microbiome.

The precise manner in which exercise influences the composition of the gut microbiome remains uncertain. Nevertheless, several scholars have put forth potential mechanisms. For instance, the gut-associated lymphoid tissues are populated with immune cells, and exercise has been demonstrated to modulate gene expression and Favor anti-inflammatory and antioxidant profiles [315,316]. This could potentially influence the host–microbiota interactions [311]. Additionally, exercise can affect the mucus layer in the gut, which serves as a vital barrier separating microbes from the gut lining. Moreover, exercise affects the motility of the gut, which can influence the GI transit time. Consequently, this can result in alterations to microbial habitats and their access to nutrients. Additionally, physical activity has been linked to alterations in bile acid circulation, which plays a pivotal role in regulating the structure of the microbial community [317]. Moreover, exercise increases metabolic demands, which results in the release of compounds such as lactate and myokines. These substances can interact with the gut environment [311]. The combination of these potential mechanisms is likely to mediate the impact of exercise on the gut microbiota. Nevertheless, there is still considerable uncertainty regarding the magnitude and consistency of these effects. Moreover, there is a dearth of research in this area. As researchers continue to investigate the impact of exercise on the gut microbiome, new potential mechanisms will emerge.

A recent systematic review by Boytar et al. has indicated that moderate to high-intensity exercise for 30–90 min, performed on more than two days per week for a minimum of eight weeks, may result in alterations to the gut microbiota. The review included 20 studies, encompassing both healthy and clinical populations. The authors’ overall findings indicated that exercise has a beneficial impact on the gut microbiota in both healthy and clinical populations [318]. Furthermore, the integration of exercise with dietary interventions has been demonstrated to enhance the composition of the gut microbiota. For instance, in a study by Cronin et al. (2018), participants engaged in a combined regimen of aerobic and resistance training three times a week for eight weeks while consuming 24 g of protein. The intervention resulted in a lower archaeal Shannon’s index but a higher bacterial Shannon’s index compared to the group that only consumed protein [319]. Furthermore, the intensity of the exercise also influences the change in microbial composition. In their study, Torquati et al. evaluated participants who either performed moderate-intensity exercise or high-intensity exercise. The post-exercise alpha diversity was found to differ significantly between the groups (*p* < 0.05). Following exercise, the relative abundance of Bifidobacterium and butyrate-producing bacteria, including Lachnospira eligens and Enterococcus spp., was observed to be greater at lower exercise intensities [320]. Additionally, several studies have failed to observe any change in the gut microbiome with exercise [321,322].

Exercise may also influence the gut microbiome-derived metabolites. In their study, Liu et al. reported that a 12-week high-intensity combined aerobic and resistance training program in overweight and obese males led to a reduction in both faecal and serum levels of branched-chain amino acids and aromatic amino acids. Moreover, an increase in faecal propionate, GABA, and serum SCFAs was observed among those who demonstrated a positive response to the exercise regimen [323]. Although the research on the impact of exercise on gut microbiota is still in its infancy, a significant amount of evidence has emerged that identifies the beneficial impact of exercise on gut microbiota. Nevertheless, further research is required to elucidate the underlying mechanisms by which exercise influences gut microbial diversity.

### 4.3. Stress

Stress is defined as the response of an organism to a challenging situation that disrupts the balance between different elements and affects the stability of its internal environment. This response involves various molecular events that result in cellular stress [324]. The HPA axis exhibits a strong reactivity to psychosocial stress. The stimulation of the HPA axis leads to an increase in the presence of glucocorticoids in the bloodstream, with the highest levels of cortisol in the plasma occurring around 15 min after the onset of the stressor. Furthermore, the impact of the autonomic nervous system is influenced by physical and environmental stressors, as well as social stress. The sympathetic nervous system becomes more active, while the parasympathetic nervous system becomes less active, resulting in alterations to heart rate and heart rate variability. This phenomenon is known as parasympathetic withdrawal [325].

The mounting evidence indicates that the structure of the GI microbiota plays a pivotal role in both stress-related illnesses and stress resilience. It is established that Lactobacillus enhances stress resilience, and the DR 5-HT system is likely to play a significant role in this process. The corticosterone levels and anxiety-like behaviour in stress-susceptible mice were reduced when they were administered Lactobacillus murinus orally for a period of two weeks. The mRNA levels of tryptophan hydroxylase 2, an enzyme that controls the rate of serotonin synthesis, were found to be considerably higher in mice that are prone to stress [326]. Psychological stress increases the likelihood of developing a peptic ulcer and increases *Helicobacter pylori* in the stomach lining while also causing an increase in corticosterone levels in both humans and mice. Conditions such as having separated or divorced parents, experiencing conflict with parents, experiencing multiple psychological traumas, staying up past 11 p.m., having irregular eating habits, avoiding drinking water, being exposed to heat, and maternal separation are all associated with a greater likelihood of developing a peptic ulcer [327]. The disruption of circadian rhythms can cause an imbalance in the functioning and structure of the body’s natural processes, which can increase the likelihood of the development of pathogenic mechanisms. Given the current state of civilization, which promotes the disruption of natural sleep–wake cycles through extended work schedules, uncontrolled eating habits, and excessive exposure to light pollution [328].

Studies have confirmed that stress affects neuroendocrine and neuroimmune pathways, which in turn affects the likelihood of experiencing physical and mental health problems through the MGBA mechanism in humans and animals [329]. As a result, social disruption has decreased bacterial diversity [330] and *Lactobacillus* abundance [331] in mice. Additionally, chronic stress has increased the abundance of harmful bacteria from families such as *Helicobacter*, *Peptostreptococcaceae*, *Streptococcus*, *Enterococcus faecalis*, and *Akkermansia*, and decreased beneficial bacteria from families such as *Rikenella*, *Roseburia*, *Lachnospiraceae*, and *Lactobacillus* in mice [332,333]. Chronic stress induced by test preparation and academic testing was associated with a decrease in beneficial Bifidobacterium, intestinal lactic acid bacteria, and an increase in *Streptococcus* spp. [334,335]. Stressful life events in the past year were associated with a lower abundance of alpha diversity, *Firmicutes*, and *Phascolarctobacterium* and increased *Bacteroides*, *Parabacteroides*, *Rhodococcus*, and *Methanobrevibacter* [336]. While perceived stress was associated with a lower abundance of *Firmicutes*, *Anaerostipes*, and *Eubacterium* and a higher abundance of *Parabacteroides* [337,338]. Early life stress in animals impacted the abundance and ratio of *Firmicutes*, *Bacteroidetes*, *Akkermansia*, *Flexibacter*, *Prevotella*, *Lachnospiraceae*, *Porphyromonadaceae*, *Bacteroides*, *Lactobacillus*, *Alloprevotella*, *Mucispirillum*, *Desulfovibrio*, *Fusobacterium*, *Bacteroides genus*, *Streptococcus*, *Staphylococcus*, and *Sporobacter* in line with increased proinflammatory biomarkers like hippocampal IL-1β [339,340,341,342,343,344]. On the other hand, early lifetime trauma in humans, resulted in decreased *Actinobacteria*, *Lentisphaerae*, *Verrucomicrobia*, 5-oxoproline, malate, urate, and glutamate gamma methyl ester [345,346].

In utero stress in humans was associated with elevated Erwinia, Haemophilus, Serratia and lower Slackia, Actinobaculum, Paraprevotella, Butyricimonas, Citrobacter, Ruminococcus, Phascolarctobacter, Anaerotruncus, Enterococcus, and Lactobacillus [347,348]. Finally, institutional care was related to lower alpha diversity, Lachnospiraceae and an increased abundance of Prevotella, Bacteroides, Coprococcus, Streptococcus, and Escherichia, in comparison with non-institutionalized adolescents [349,350]. Collectively, data from animal and human studies indicate that various forms of stress affect the gut microbiota, which is deeply intertwined with the neuroendocrine-neuroimmune axis.

### 4.4. Genetics and Epigenetics

#### Evidence on the Genetic Links between GI and Neuropsychiatric Conditions: Common Genetic Determinants to Common Therapies?

As previously demonstrated in this review and in other studies [351], the evidence on the correlations between GI disorders and neuropsychiatric disorders is overwhelming, with clear implications for the gut–brain axis. Consequently, genetic links have been suspected between some pairs of the two types of disorders, and in fact, have been demonstrated using genome-wide association studies and linkage disequilibrium score regression [352,353,354]. For instance, Wu et al. (2021) [354] demonstrated genetic similarity across several GI diseases, including peptic ulcer disease (PUD), gastroesophageal reflux disease (GERD), and irritable bowel syndrome (IBS), with psychiatric disorders, with a particular focus on major depression. Furthermore, significant positive SNP-based genetic correlations (rg) have been observed between PUD, GORD, IBS, depressive symptoms, neuroticism [355], major depression [356], ADHD [357], and insomnia [358,359]. Pouget et al. (2019) employed GWAS data for SCZ and 19 immune diseases, including some GI autoimmune conditions, and identified genetic variation at rs1734907 that modulates the risk of SCZ and Crohn’s disease via the altered methylation and expression of EPHB4. They also observed genetic correlations between SCZ and IBS, Crohn’s disease, ulcerative colitis, and primary biliary cirrhosis [360].

More recently, a pleiotropic analysis of large-scale genome-wide association studies of 24 pairs comprising four GI diseases (inflammatory bowel disease, IBS, PUD, and GORD) and six neuropsychiatric disorders (SCZ, bipolar disorder, MDD, ADHD, posttraumatic stress disorder, and anorexia nervosa) revealed significant common genetic determinants between 22 of the 24 evaluated GI-psychiatric conditions trait pairs. Of these, 19 pairs showed 2910 significant pleiotropic single nucleotide variants (SNVs) under a composite null hypothesis [361]. A significant number of common genetic determinants were identified between 22 of the 24 evaluated GI-psychiatric conditions trait pairs. Of these, 19 pairs were found to potentially exhibit 2910 significant pleiotropic single nucleotide variants (SNVs) under a composite null hypothesis [361]. Furthermore, this led to the identification of 83 pleiotropic loci, 24 colocalized loci, and 158 unique candidate pleiotropic genes. Notably, several of the identified pleiotropic loci share causal variants with gut microbiomes, and many of those loci are common among multiple trait pairs. Several trait pairs were identified, including those involving the loci on 1q32.1 (*INAVA*), 19q13.33 (*FUT2*), 11q23.2 (*NCAM1*), and 1p32.3 (*LRP8*). For instance, variants in the FUT2 gene are shared between peptic ulcer disease, SCZ, and ADHD. The associations between peptic ulcer and ADHD, however, are variant-dependent.

Despite the significant efforts and progress that have been made, several questions remain unanswered. These include whether the genetic links indicate shared susceptibility genes between the two types of phenotypes [362,363] or whether the relationships between the pairs are of the causal types [364,365]. Another crucial question that has emerged is whether it is feasible to simultaneously target those disease pairs for treatment or intervention.

## 5. Conclusions

The gut microbiota is susceptible to direct intervention through the administration of prebiotics, probiotics, and antibiotics, and its composition can be influenced by lifestyle factors. Although the relationship between the gut microbiota and neurodegenerative diseases is currently a subject of intense investigation, there is a need to develop new techniques to elucidate the communication between neurological diseases and the gut microbiota. In this study, we concentrate on the pathogenesis of NDDs and the factors influencing the formation of the gut microbiota. It is therefore reasonable to hypothesize that drugs used to treat these conditions may act, at least in part, by modifying the gut microbiome, which could be a potential therapeutic target. By understanding the MGBA, it may be possible to facilitate research into microbial-based interventions and therapeutic strategies for neurological diseases.

## 6. Future and Prospects

The experimental data presented in this review provide substantiation for the hypothesis that disturbances in the composition of the microbiota are a contributing factor in the development of neurological disorders. Given that the gut microbiota undergoes modifications even during foetal development, elucidating the mechanisms of communication between the gut and the brain has a profound impact on the development of novel therapeutic strategies. Furthermore, additional research is necessary to investigate the efficacy of probiotics in individuals with neurological disorders. In the future, the ability to sequence the entire microbiome in patients with neurological disorders will pave the way for a probiotic-based therapy, which will help to prevent the progression of the disease.

## Figures and Tables

**Table 1:** The relationship between gut bacterial composition and Rett syndrome. Abbreviations: multiple species of the same genus (spp.).

Condition	Bacteria Phylum	Genus/spp.	Condition Compared to Control Group	References
Rett Syndrome	Firmicutes	*Ruminococcus* spp.	↓ levels in the intestinal microbiome	[26,29,30]
Rett Syndrome	Firmicutes	*Faecalibacterium prausnitzii (species)*	↓ levels in the intestinal microbiome	[26,29,30]
Rett Syndrome	Firmicutes	*Clostridium* spp.	↑ levels in the intestinal microbiome	[26,29,30]
Rett Syndrome	Firmicutes	*Sutterella* spp.	↑ levels in the intestinal microbiome	[26]
Rett Syndrome	Firmicutes	*Erysipelatoclostridium*	↑ levels in the intestinal microbiome	[26,29]
Rett Syndrome	Firmicutes	*Lactobacillaceae* spp.	↑ levels in the intestinal microbiome	[29,30]
Rett Syndrome	Firmicutes	*Oscillibacter* spp.	↓ levels in the intestinal microbiome	[30]
Rett Syndrome	Firmicutes	*Sporobacter* spp.	↓ levels in the intestinal microbiome	[30]
Rett Syndrome	Firmicutes	*Veillonellaceae* spp.	↑ levels in the intestinal microbiome	[31]
Rett Syndrome	Firmicutes	*Enterococcus* spp.	↑ levels in the intestinal microbiome	[29]
Rett Syndrome	Bacteroidetes	*Bacteroides* spp.	↑ levels in the intestinal microbiome	[26,29]
Rett Syndrome	Bacteroidetes	*Prevotella* spp.	↓ levels in the intestinal microbiome	[26,29,30]
Rett Syndrome	Bacteroidetes	*Barnesiella* spp.	↓ levels in the intestinal microbiome	[30]
Rett Syndrome	Bacteroidetes	*Alistipes* spp.	↓ levels in the intestinal microbiome	[30]
Rett Syndrome	Bacteroidetes	*Odoribacter* spp.	↓ levels in the intestinal microbiome	[31]
Rett Syndrome	Bacteroidetes	*Butyricimonas* spp.	↓ levels in the intestinal microbiome	[31]
Rett Syndrome	Bacteroidetes	*Rikenellaceae* spp.	↑ levels in the intestinal microbiome	[31]
Rett Syndrome	Actinobacteria	*Bifidobacterium* spp.	↑ levels in the intestinal microbiome	[26,29,30]
Rett Syndrome	Actinobacteria	*Actinomyces* spp.	↑ levels in the intestinal microbiome	[26,29]
Rett Syndrome	Actinobacteria	*Eggerthella* spp.	↑ levels in the intestinal microbiome	[29]
Rett Syndrome	Proteobacteria	*Escherichia/Shigella* spp.	↑ levels in the intestinal microbiome	[26,29]
Rett Syndrome	Verrucomicrobia	*Verrucomicrobiaceae* spp.	↓ levels in the intestinal microbiome	[31]

**Table 2:** Investigating the gut microbiota composition of individuals with ADHD. Abbreviations: attention-deficit/hyperactivity disorder (ADHD), multiple species of the same genus (spp.), beta diversity (β-diversity), alpha diversity (α-diversity), Schedule for Affective Disorders and Schizophrenia for School-Age Children-Present and Lifetime version (K-SADS-PL), complex regional pain syndrome (CPRS), Diagnostic and Statistical Manual of Mental Disorders, 4th ed. (DSM-IV), Linear Discriminant Analysis Effect Size (LEfSe), 16S ribosomal RNA (or 16S rRNA), Cluster of Differentiation 74 (CD74), and tumour necrosis factor (TNF).

Assessment Methods	Potential Confounders	Taxonomic Composition Changes	Potential Changes in Synaptic Plasticity	References
ADHD diagnosed using Kiddie-SADS-PL, symptoms severity assessed with CPRS	Dietary habits, gastrointestinal symptoms, depression, ADHD medications	No significant change in α or β-diversity ↓ *Faecalibacterium* levels	↑ Systematic inflammation ↑ Gut permeability ↑ Unbalanced neurotransmitters levels in the brain	[47]
Diagnosed using DSM-IV via K-SADS, symptom severity assessed with CPRS.	ADHD medications	↑ β-diversity in ADHD ↑ *Ruminococcaceae_UGC_004*	CD74, TNF, cytokine receptors	[48]
Wilcoxon tests for species abundance, LEfSe method for taxa differences, symptom severity assessed with CPRS	Gastrointestinal symptoms, depression or anxiety, use of probiotics or antibiotics, obesity, allergy	↓ *Faecalibacterium prausnitzii* *↓ Lachnospiraceae* spp. *↓ Ruminococcus gnavus* *↑ Bacteroides caccae* *↑ Odoribacter* *Splanchnicus* *↑ Paraprevotella* *Xylaniphila* *↑ Veillonella parvula*	↑ Inflammatory factors ↓ Neuroplasticity	[49]
Children’s Global Assessment Scale, ADHD Rating Scale-IV, 16S rRNA gene sequencing, dietary intake questionnaire	Unspecified	No significant change in α or β-diversity ↓ *Bifidobacterium*	↑ Neural signalling modulation ↓ Inflammatory responses ↓ Gut–brain axis dysregulation ↓ Oxidative stress levels	[50]

**Table 3:** List of bacteria involved in Parkinson’s disease. Abbreviations: multiple species of the same genus (spp.).

Condition	Bacteria Phylum	Genus/spp.	Condition Compared to Control Group	References
Parkinson’s Disease	Verrucomicrobia	*Akkermansia* spp.	↑ levels in the intestinal microbiome	[124,127,128,130]
Parkinson’s Disease	Firmicutes	*Blautia* spp.	↓ levels in the intestinal microbiome	[129]
Parkinson’s Disease	Firmicutes	*Coprococcus* spp.	↓ levels in the intestinal microbiome	[129]
Parkinson’s Disease	Firmicutes	*Roseburia* spp.	↓ levels in the intestinal microbiome	[129]
Parkinson’s Disease	Firmicutes	*Faecalibacterium* spp.	↓ levels in the intestinal microbiome	[123,129]
Parkinson’s Disease	Proteobacteria	*Ralstonia* spp.	↑ levels in the intestinal microbiome	[129]
Parkinson’s Disease	Proteobacteria	*Enterobacteriaceae* spp.	↑ levels in the intestinal microbiome	[122]
Parkinson’s Disease	Bacteroidetes	*Prevotellaceae* spp.	↓ levels in the intestinal microbiome	[122]

**Table 4:** Relationship between gut microbiota and temporal lobe epilepsy. Abbreviations: levodopa (L-Dopa), spike-timing-dependent plasticity (STDP), 5-hydroxytryptophan (5-HTP).

Neurotransmitters	Precursors	Gut Microbiota	Proposed Roles within the Gut–Brain Axis	References
Glutamate	Acetate	*Lactobacillus plantarum* *Bacteroides vulgatus* *Campylobacter jejuni*	Transfer intestinal sensory signals to the brain through the vagus nerve; regulate neurogenesis; synaptogenesis; neuron survival	[190]
Acetylcholine	Choline	*Lactobacillus plantarum* *Bacillus acetylcholine* *Bacillus subtilis* *Escherichia coli* *Staphylococcus aureus*	Regulate intestinal motility and secretion and enteric neurotransmission; retain brain plasticity	[191]
Dopamine	Tyrosine L-DOPA	*Staphylococcus*	Promote intestinal motility and modulates STDP	[192]
Serotonin	5-HTP Tryptophan	*Staphylococcus* *Clostridial* spp.	Remodels neuronal cytoarchitecture	[193]
GABA	Acetate	*Bifidobacterium* *Bacteroides fragilis* *Parabacteroides* *Eubacterium*	Modulates synaptic transmission in the ENS; regulates inhibitory–excitatory balance	[194,195]

## Data Availability

Not applicable.

## Abbreviations

AD	Alzheimer’s disease
ADHD	Attention-deficit/hyperactivity disorder
ALS	Amyotrophic lateral sclerosis
APOE	Apolipoprotein E
ASD	Autism spectrum disorder
ASMs	Antiseizure medications
BBB	Blood–brain barrier
BDNF	Brain-derived neurotrophic factor
CJD	Creutzfeldt–Jakob disease
CNS	Central nervous system
C-sections	Caesarean section
ENS	Enteric nervous system
FMT	Faecal microbiota transplantation
FTLD	Fronto-temporal lobe Dementia
GABA	Gamma-aminobutyric acid
GF	Germ-free
GI	Gastrointestinal
HPA-axis	Hypothalamic-pituitary-adrenal axis
IL	Interleukin
MDD	Major depressive disorder
MGBA	Microbiota–gut–brain axis
MHC	Major histocompatibility complex
MS	Multiple sclerosis
MSA	Multisystem atrophy
NDDs	Neurodegenerative diseases
PD	Parkinson’s disease
PFC	Prefrontal cortex
SCFAs	Short-chain fatty acids
SCZ	Schizophrenia
SGA	Second-generation antipsychotics
TLE	Temporal lobe epilepsy
WD	Wilson–Konovalov disease
